# Assessment of potential invasion for six phytophagous quarantine pests in Taiwan

**DOI:** 10.1038/s41598-021-89914-w

**Published:** 2021-05-21

**Authors:** Hsin-Ting Yeh, Harn-Yeu Cheah, Ming-Chih Chiu, Jhih-Rong Liao, Chiun-Cheng Ko

**Affiliations:** 1 The Experimental Forest, College of Bio-Resources and Agriculture, National Taiwan University, Zhushan Township, Nantou County, 557004 Taiwan; 2 Master Program for Plant Medicine, College of Bio-Resources and Agriculture, National Taiwan University, Taipei City, 106332 Taiwan; 3 Center for Marine Environmental Studies (CMES), Ehime University, Matsuyama, Ehime, 7908577 Japan; 4 Department of Entomology, National Taiwan University, Taipei City, 106332 Taiwan

**Keywords:** Ecology, Agroecology, Biogeography, Ecological modelling, Invasive species

## Abstract

Pest risk assessment is typically performed by expert taxonomists using a pest’s biological data. However, the biological data or expert taxonomists may be difficult to find. Here, we used species distribution modelling to predict potential invasion in which phytophagous quarantine pests survive in Taiwan; the pests (unrecorded yet in Taiwan) included were three notorious quarantine whiteflies (*Crenidorsum aroidephagus*, *Aleurothrixus trachoides*, and *Paraleyrodes minei*) and three aphids (*Nasonovia ribisnigri*, *Macrosiphum euphorbiae*, and *Viteus vitifoliae*). In brief, maximum entropy modelling (MaxEnt) was used to predict the suitability of the pests’ habitats under certain climatic conditions, and then receiver operating characteristic curve analysis was performed (to verify the prediction result). We then analysed environmental variables affecting the habitat suitability and matched them with Taiwan’s crop cultivation areas for the assessment of potential invasion. We observed that the habitat suitability of the cultivation areas of host plants was low for *C. aroidephagus*, *A. trachoides*, and *N. ribisnigri* but was high for the remaining three species. Moreover, precipitation of coldest quarter negatively affected habitat suitability for *C. aroidephagus*, *P. minei*, *N. ribisnigri*, and *M. euphorbiae.* Seasonal temperature changes also negatively affected the habitat suitability for *A. trachoides*. This is the first study to demonstrate the use of species distribution modelling as the preliminary step for the pest risk assessment of these emerging pests with limited biological data before their invasion.

## Introduction

Biological invasion is a serious global economic and ecological threat^1,2^. Invasive organisms negatively influence native fauna and flora through multiple interactions, such as predation or competition^3,4^. Multiple human activities (e.g., international trade, travel) have promoted the movement of alien species across geographical barriers^5^. Therefore, interruption of pest invasion by plant quarantine is crucial topic related to biological invasion. For the management of alien species invasion, pest risk assessment can be used to evaluate the probability of invasion and spread of a pest. It should entail the use of scientific methods to determine whether government intervention is necessary in the prevention of pest invasion, and can also be used to assess the potential effects of an invasive species. Pest risk assessment includes identification of transmission route and biological characteristics and review of previous risk analysis results, and records of invasion of other countries^6^. It is typically performed by expert taxonomists. However, due to the wide abundance of insects, not every insect’s biological data is available. Even experienced experts sometimes have difficulty obtaining sufficient data for risk assessment. Therefore, this study used species distribution modelling (SDM) to assess potential invasions; this modelling approach is useful under the condition of limited biological data and provides greater insight into the potential distribution (or habitat suitability) than specialist knowledge alone can.

SDM, also known as ecological niche modelling refers to mathematical statistics or prediction modules based on the ecological niche theory^7^. SDM helps to understand the species niche or environmental space on the basis of known species’ distribution data and environmental characteristics^8^. It can also be used to predict habitat suitability before invasion by alien species^9^, and also aids in early monitoring, warning generation, and emergency control measure adoption. Maximum entropy modelling (MaxEnt) is a machine learning method based on presence-only data, devised by Phillips et al.^8^. This method is widely used because it requires a small sample size and provides high prediction accuracy and its software easy to use^10,11^. The model’s predictive ability and the use of normalisation prevent over-fitting during predictive simulation^8,12^. Zhang et al.^13^ analysed potential areas for invasion by the pest *Viteus vitifoliae* (Fitch) in China using MaxEnt and geographic information system (GIS) and proposed immediate preventive measures. Prabhulinga et al.^14^ predicted the habitat suitability of *Bemisia tabaci* (Gennadius) in North India using MaxEnt. MaxEnt is a crucial tool not only for understanding relationships between biological habitat and the related environmental factors but also for managing invasive alien species^9^.

Whiteflies (superfamily Aleyrodoidea) and aphids (superfamily Aphidoidea) both belong to the order Hemiptera, suborder Sternorrhyncha. Thus far, more than 1,660 known whiteflies^15,16^ and nearly 5,000 known aphids have been recorded worldwide^17–19^. Both small phytophagous insects with piercing and sucking mouthparts; certain species can transmit plant disease and become notorious agricultural and forestry pests. International trade, tourism, and business activities have increased year by year. The invasion by alien pests is greatly raised by man-made activities (e.g., importing agricultural products, mail packages, passenger luggage) and even affects agricultural production and the ecological environment^5^. For example, the sweet potato whitefly, *Bemisia tabaci* (MAMAI, b-biotype) invaded Taiwan in the 1990s and brought huge economic losses^20^.

**Figure 1:**
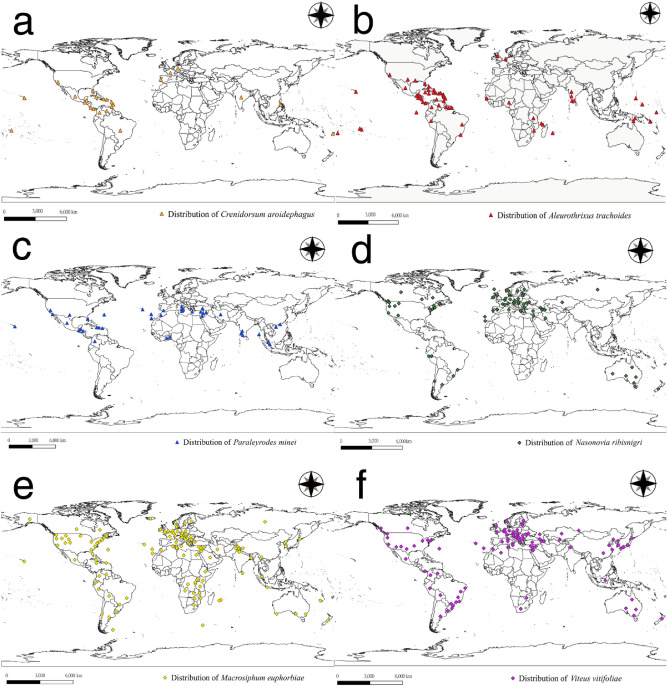
Distribution data of six important quarantine species: (**a**) *Crenidorsum aroidephagus*, (**b**) *Aleurothrixus trachoides*, (**c**) *Paraleyrodes minei*, (**d**) *Nasonovia ribisnigri*, (**e**) *Macrosiphum euphorbiae*, (**f**) *Viteus vitifoliae*. Triangle represent three whiteflies’ distribution points; diamond shapes represent three aphids’ distribution points. Maps were created using QGIS version 3.12.1 (www.qgis.org).

The present study aimed to establish a model for predicting the habitat suitability of different areas for quarantine pests before their invasion. The study results can be used by the government as a reference to formulate strategies for pest quarantine and monitoring. According to plant quarantine records in Taiwan (data obtained from the Bureau of Animal and Plant Health Inspection and Quarantine, Council of Agriculture, Executive Yuan) and the pest list of Centre for Agriculture and Bioscience International (CABI) and Invasive Species Compendium, six prominent quarantine pests have not yet been recorded in Taiwan, namely three whiteflies [*Crenidorsum aroidephagus* Martin & Aguiar, *Aleurothrixus trachoides* (Back), and *Paraleyrodes minei* Iaccarino] and three aphid species [*Nasonovia ribisnigri* (Mosley), *Macrosiphum euphorbiae* (Thomas) and *V. vitifoliae*]. MaxEnt was used to predict habitat suitability under certain climatic conditions for these six quarantine pests based on distribution points (Fig. 1, Supplementary Tables S1–S6), which were then matched with the information on host plant cultivation areas. The result is a combination of climatic factors and host plant information for improving the credibility of risk assessment for potential invasions.

## Results

### Six quarantine pests predicted by MaxEnt in potential distribution areas

**Figure 2:**
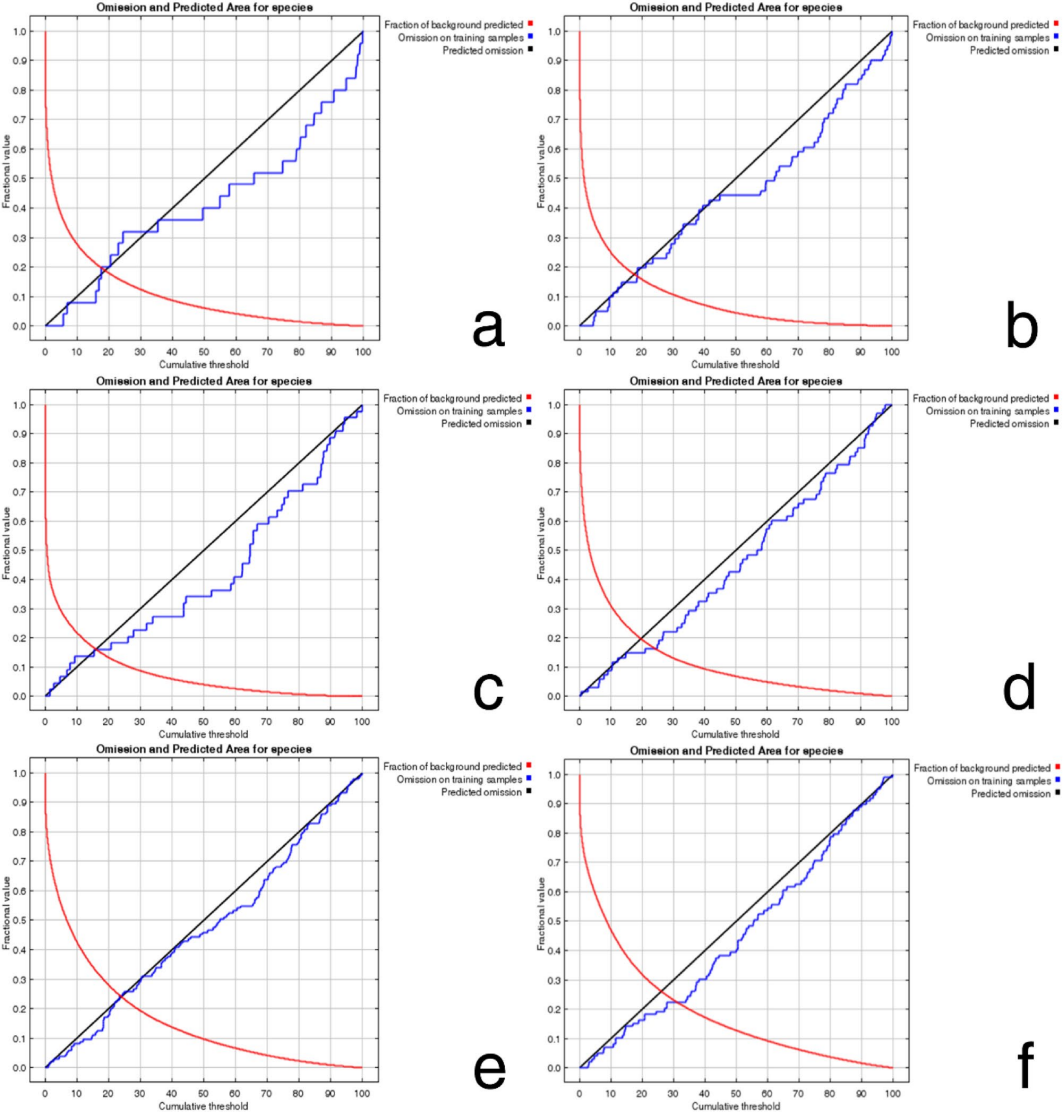
Omission rate of six important quarantine species: (**a**) *Crenidorsum aroidephagus*, (**b**) *Aleurothrixus trachoides*, (**c**) *Paraleyrodes minei*, (**d**) *Nasonovia ribisnigri*, (**e**) *Macrosiphum euphorbiae*, (**f**) *Viteus vitifoliae*. Fraction of background predicted (red line), omission rate for training samples (blue line), and predicted omission rate (black line). Figures were created using R version 4.04 (https://www.r-project.org/).

**Figure 3:**
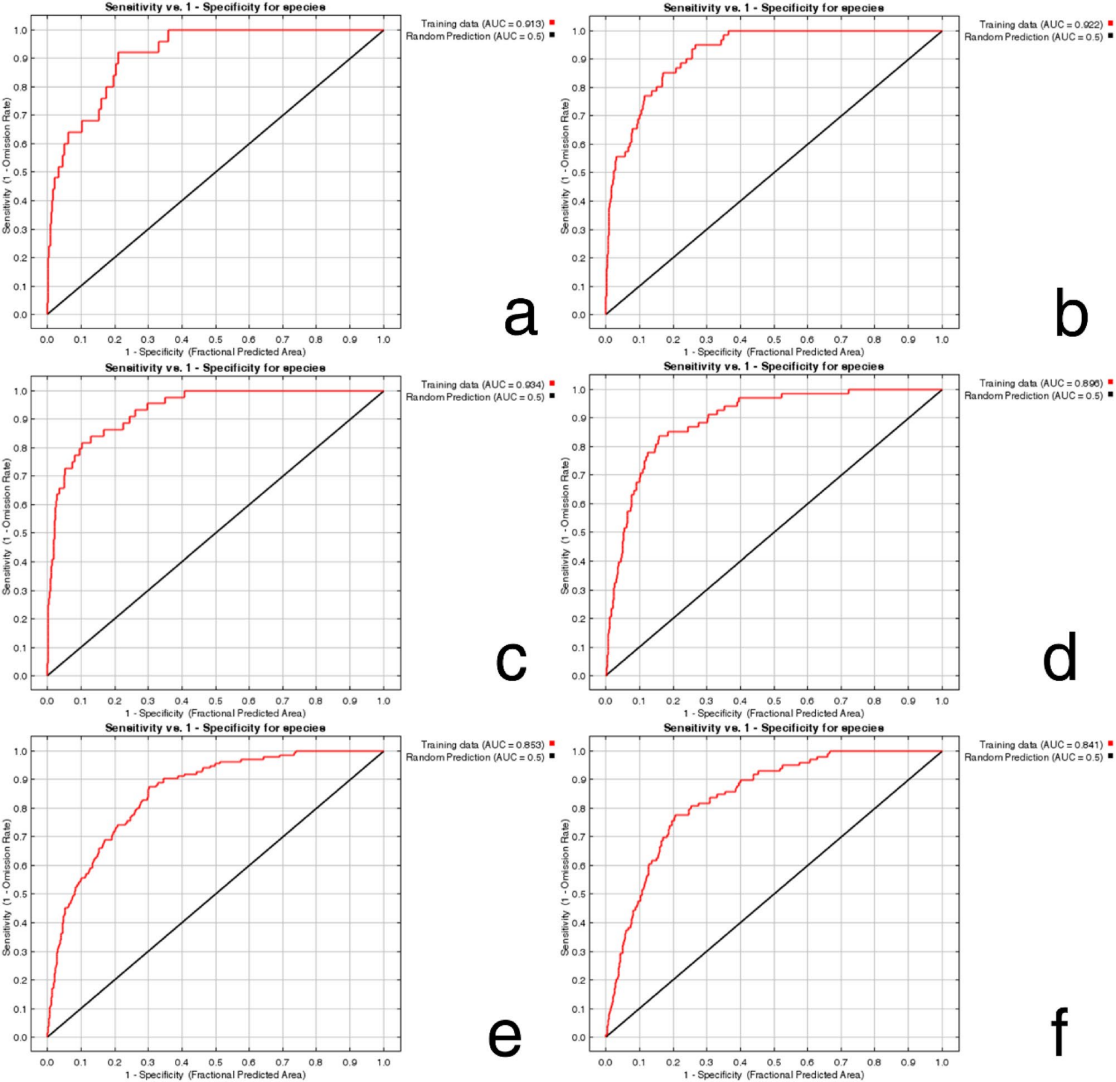
The receiver operating characteristic (ROC) curve and area under the curve (AUC) of six important quarantine species: (**a**) *Crenidorsum aroidephagus*, (**b**) *Aleurothrixus trachoides*, (**c**) *Paraleyrodes minei*, (**d**) *Nasonovia ribisnigri*, (**e**) *Macrosiphum euphorbiae*, (**f**) *Viteus vitifoliae*. Training data (red line), random prediction (black line). Figures were created using R version 4.04 (https://www.r-project.org/).

Figure 2 shows the omission rate of the predictions for the six species. The training set omission rate (blue line) should be close to the predicted omission rate (black line). The average training set omission rates for *C. aroidephagus*, *A. trachoides*, *P. minei*, *N. ribisnigri*, *M. euphorbiae*, and *V. vitifoliae* were 0.000, 0.000, 0.000, 0.015, 0.000, 0.000, respectively (all < 0.10). The areas under the receiver operating characteristic (ROC) curve (AUCs; output from the MaxEnt) of the training set for *C. aroidephagus*, *A. trachoides*, *P. minei*, *N. ribisnigri*, *M. euphorbiae*, and *V. vitifoliae* were 0.913, 0.921, 0.934, 0.896, 0.853, 0.841, respectively (Fig. 3). According to the AUC evaluation, this prediction approach achieved “moderate to high accuracy^13,21–23^.

### Comparisons of habitat suitability of important quarantine pests and crop cultivation areas

**Figure 4:**
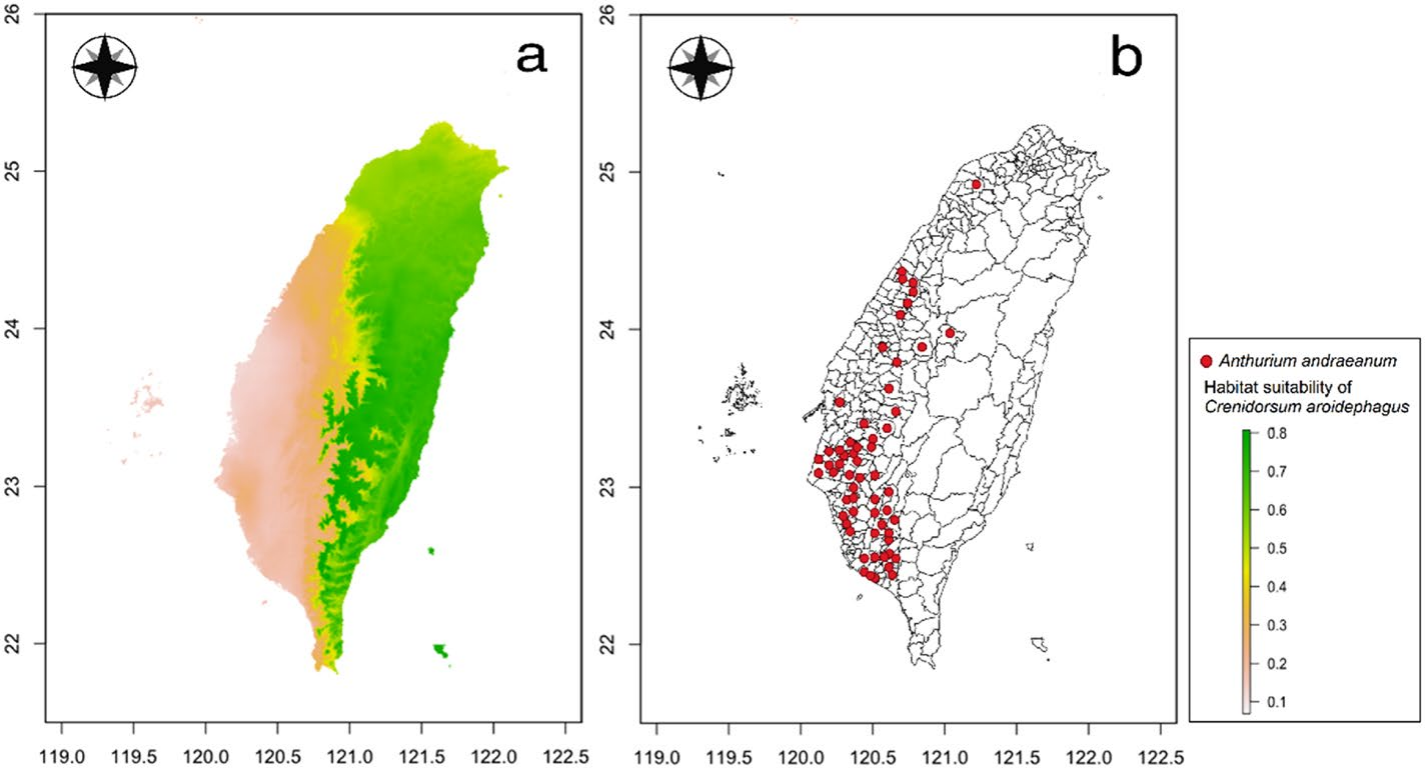
Comparison of potential distribution area of *Crenidorsum aroidephagus* and cultivation areas of *Anthurium andraeanum* in Taiwan: (**a**) potential distribution (**b**) cultivation areas. Maps were created using R version 4.04 (https://www.r-project.org/) and QGIS version 3.12.1 (www.qgis.org).

**Figure 5:**
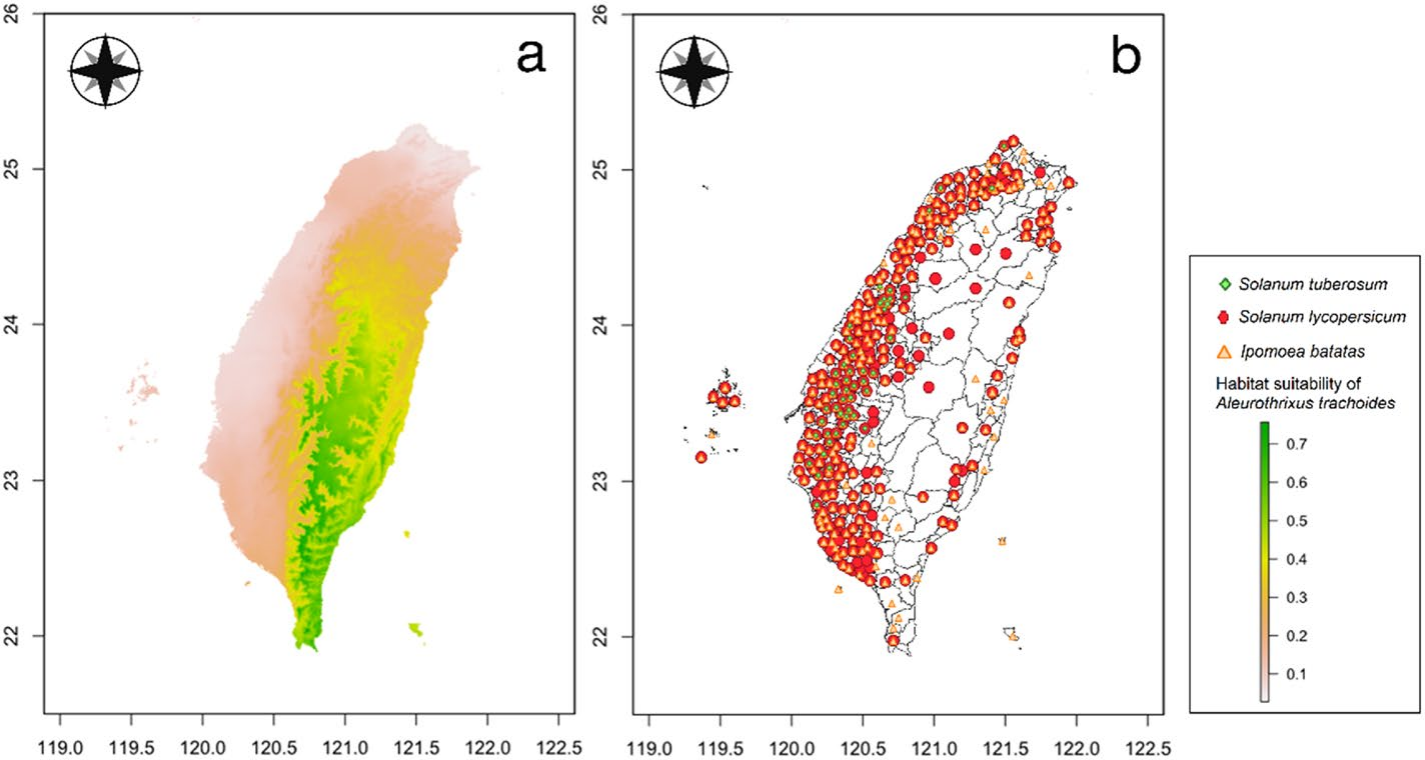
Comparison of potential distribution area of *Aleurothrixus trachoides* and cultivation area of *Solanum tuberosum*, *S. lycopersicum*, and *Ipomoea batatas* in Taiwan: (**a**) potential distribution (**b**) cultivation areas. Maps were created using R version 4.04 (https://www.r-project.org/) and QGIS version 3.12.1 (www.qgis.org).

**Figure 6:**
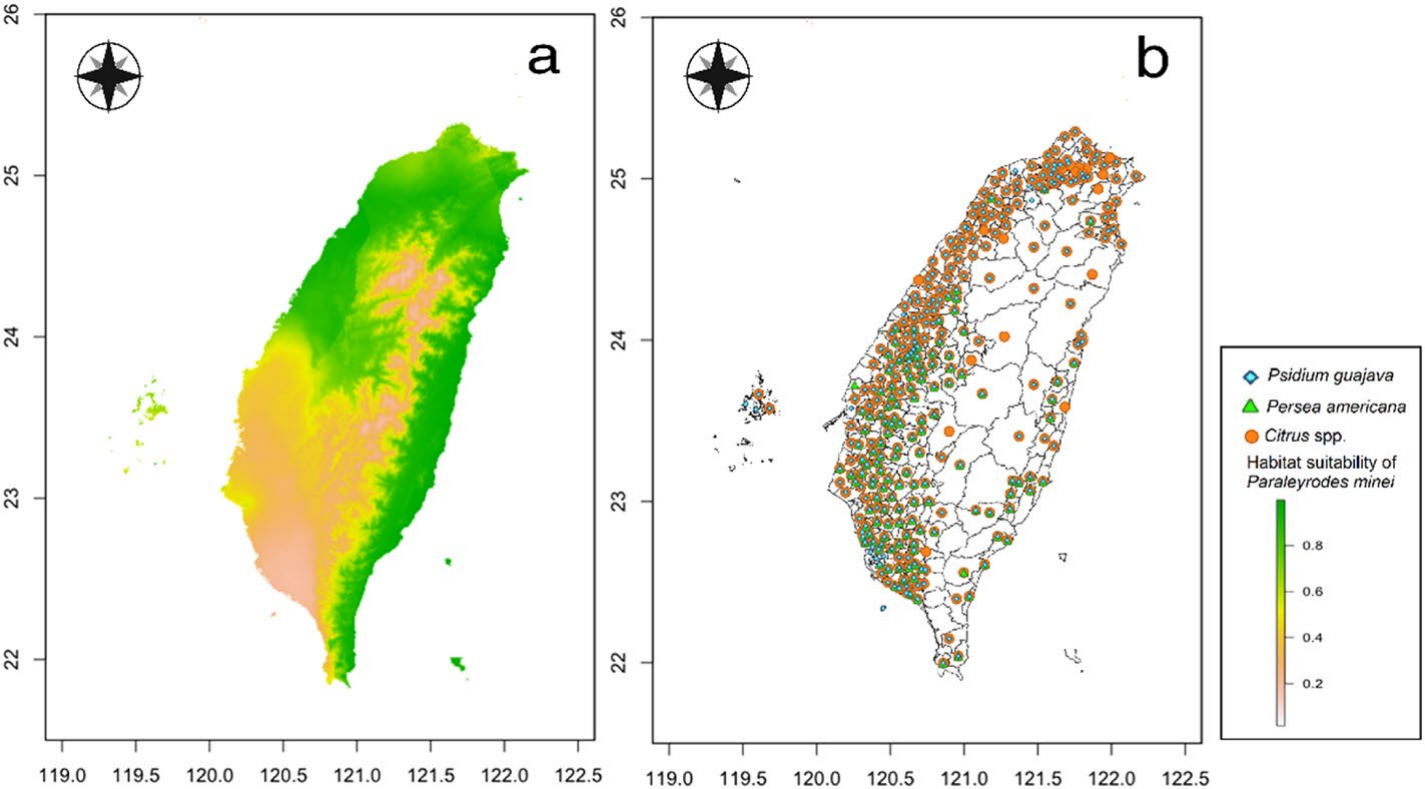
Comparison of potential distribution area of *Paraleyrodes minei* and cultivation areas of *Psidium guajava*, *Persea americana*, in Taiwan: (**a**) potential distribution (**b**) cultivation areas. Maps were created using R version 4.04 (https://www.r-project.org/) and QGIS version 3.12.1 (www.qgis.org).

**Figure 7:**
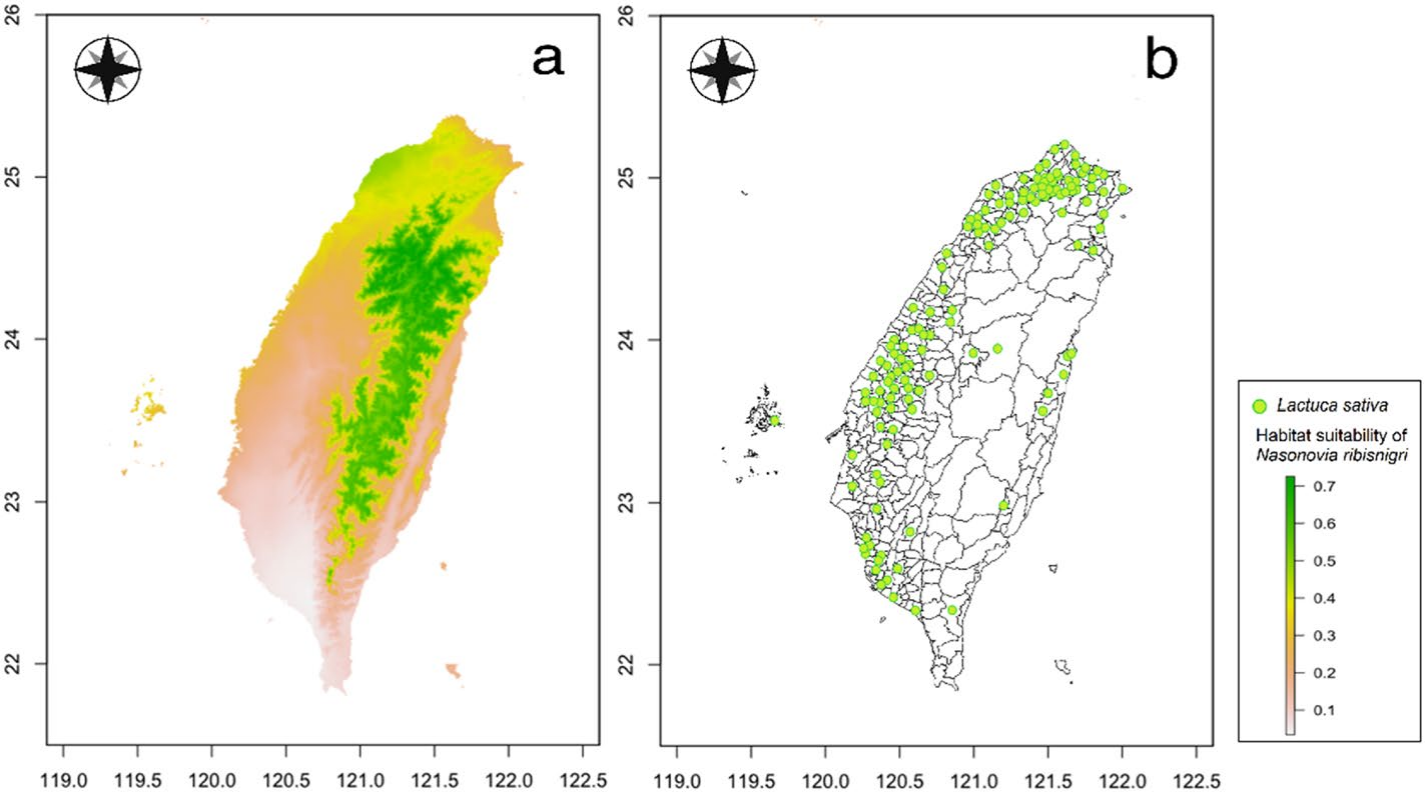
Comparison of potential distribution area of *Nasonovia ribisnigri* and cultivation areas of *Lactuca sativa* in Taiwan: (**a**) potential distribution (**b**) cultivation areas. Maps were created using R version 4.04 (https://www.r-project.org/) and QGIS version 3.12.1 (www.qgis.org).

**Figure 8:**
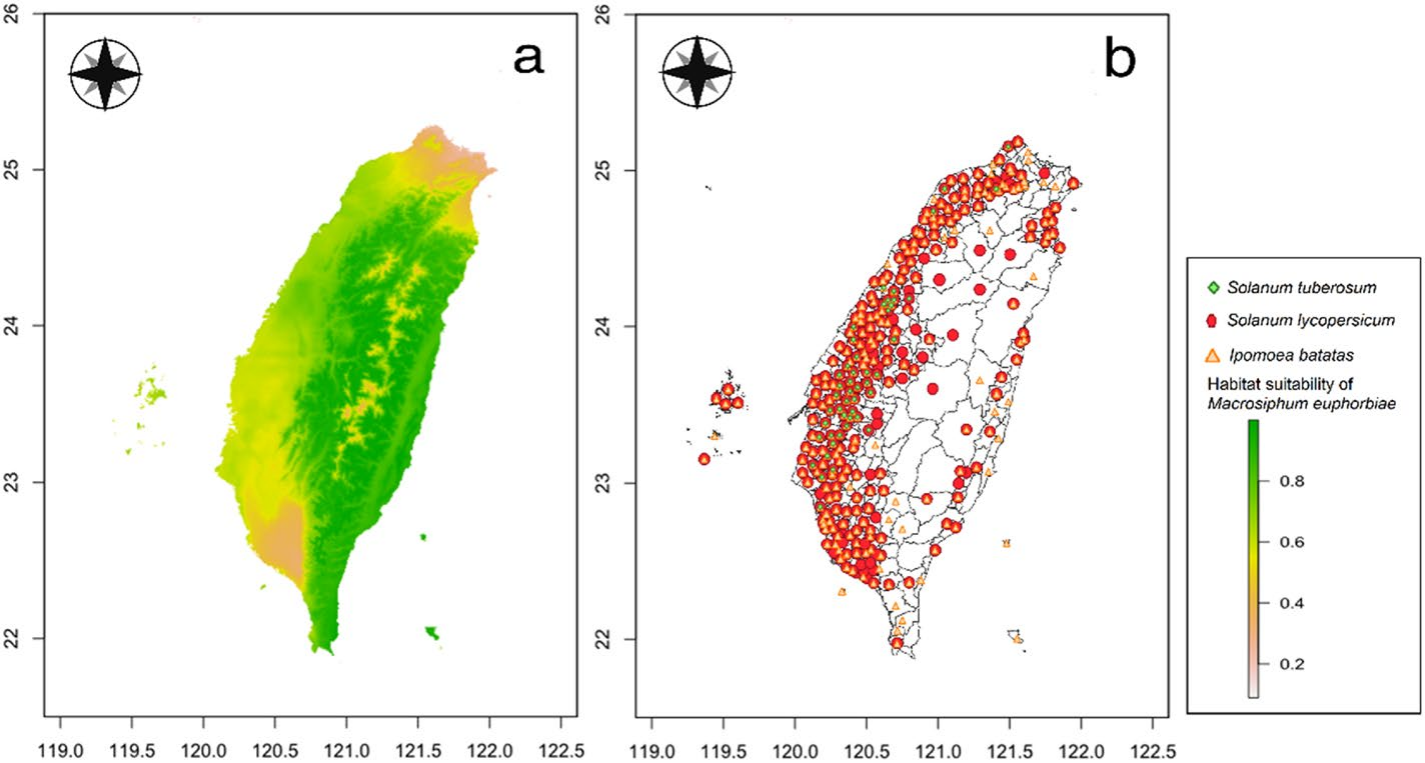
Comparison of potential distribution area of *Macrosiphum euphorbiae* and cultivation areas of *Solanum tuberosum*, *S. lycopersicum*, and *Ipomoea batatas* in Taiwan: (**a**) potential distribution (**b**) cultivation areas. Maps were created using R version 4.04 (https://www.r-project.org/) and QGIS version 3.12.1 (www.qgis.org).

**Figure 9:**
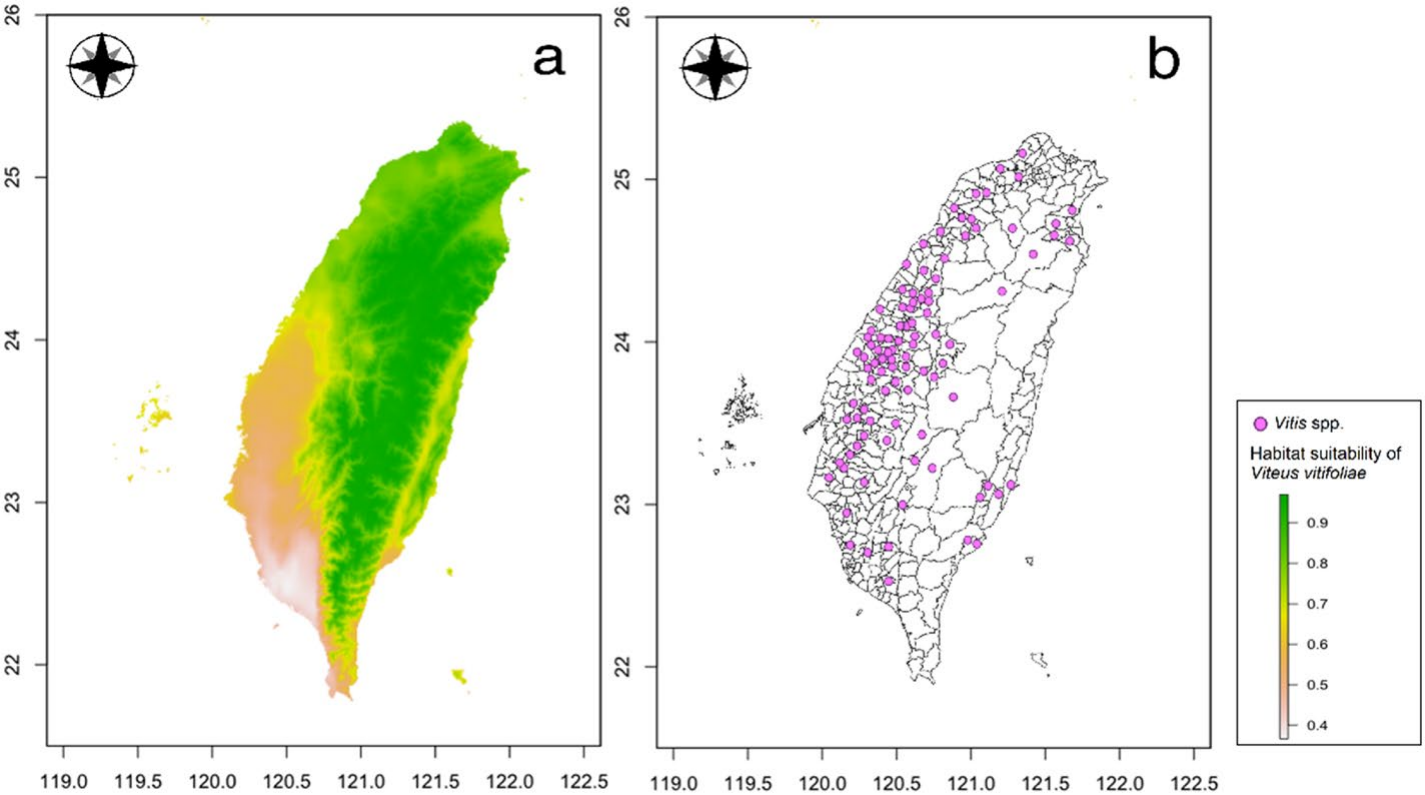
Comparison of potential distribution area of *Viteus vitifoliae* and cultivation areas of *Vitis* spp. in Taiwan: (**a**) potential distribution (**b**) cultivation areas. Maps were created using R version 4.04 (https://www.r-project.org/) and QGIS version 3.12.1 (www.qgis.org).

Comparisons of habitat suitability of six important quarantine pests in Taiwan and their host plant cultivation areas are shown in Figs. 4, 5, 6, 7, 8, and 9. Green represents high habitat suitability, red represents moderate habitat suitability, and white represents low habitat suitability. The potential distribution areas of *C. aroidephagus* are in the mid- and high-altitude mountains of Taiwan and in northern and eastern Taiwan but not in western and southwestern Taiwan. In addition, two outlying islands (Ludao and Lanyu) are also considered as highly suitable areas. The host plant *Anthurium andraeanum* of *C. aroidephagus* is mainly cultivated in western Taiwan from most plain to shallow mountainous areas (Fig. 4). The potential distribution areas of *A. trachoides* are in high-altitude mountains of Taiwan, southeastern Taiwan and in the two outlying islands of Ludao and Lanyu. The host plants potato (*Solanum tuberosum*), tomato (*S. lycopersicum*), and sweet potato (*Ipomoea batatas*) of *A. trachoides* are cultivated in most plain areas in Taiwan, particularly in western Taiwan. In addition, these crops are also cultivated in the outlying islands Penghu, Ludao, and Lanyu (Fig. 5). The potential distribution areas of *P. minei* are in most parts of Taiwan and in Ludao and Lanyu, with the exceptions being high-altitude mountainous areas and southwestern Taiwan. The host plants guava (*Psidium guajava*), avocado (*Persea americana*), and citrus (*Citrus* spp.) of *P. minei* are cultivated in plain areas in Taiwan, especially western Taiwan, and in Penghu (Fig. 6). The potential distribution areas of *N. ribisnigri* include the areas around high-altitude mountain in Taiwan. In addition, southwestern Taiwan and the mountainous region of eastern Taiwan had low habitat suitability. The host plant lettuce (*Lactuca sativa*) of *N. ribisnigri* is cultivated in most plain to shallow mountainous areas in Taiwan and Penghu (Fig. 7). The potential distribution areas of *M. euphorbiae* are in most parts of Taiwan, particularly in low- and mid-altitude mountainous areas, but not in high-altitude mountainous areas. In addition, the plain areas of northern and southwestern Taiwan are considered to have low suitability. The host plants of *M. euphorbiae* are potato, tomato and sweet potato, and the main cultivation areas are in the eastern and western plains to the shallow mountains of Taiwan (Fig. 8). The potential distribution for *V. vitifoliae* are mainly in the mid- to high-altitude mountainous areas of Taiwan and northern Taiwan, but not in southwestern Taiwan. The host plant of *V. vitifoliae* is mainly cultivated in the shallow mountainous areas to mid-altitude mountainous areas of western Taiwan, and a small part is in the southeastern region of Taiwan (Fig. 9). In addition, a histogram of habitat suitability of crop locations in six important quarantine species were shown in Supplementary Fig. S2. For example, less than 50% crop locations have a habitat suitability of > 0.5 for *C. aroidephagus*, *A. trachoides*, and *N. ribisnigri*; whereas more than 50% of crop locations have a habitat suitability of > 0.5 for *P. minei*, *M. euphrobiae*, and *V. vitifoliae*.

### Environmental variables influencing the habitat suitability of six important quarantine species

**Figure 10:**
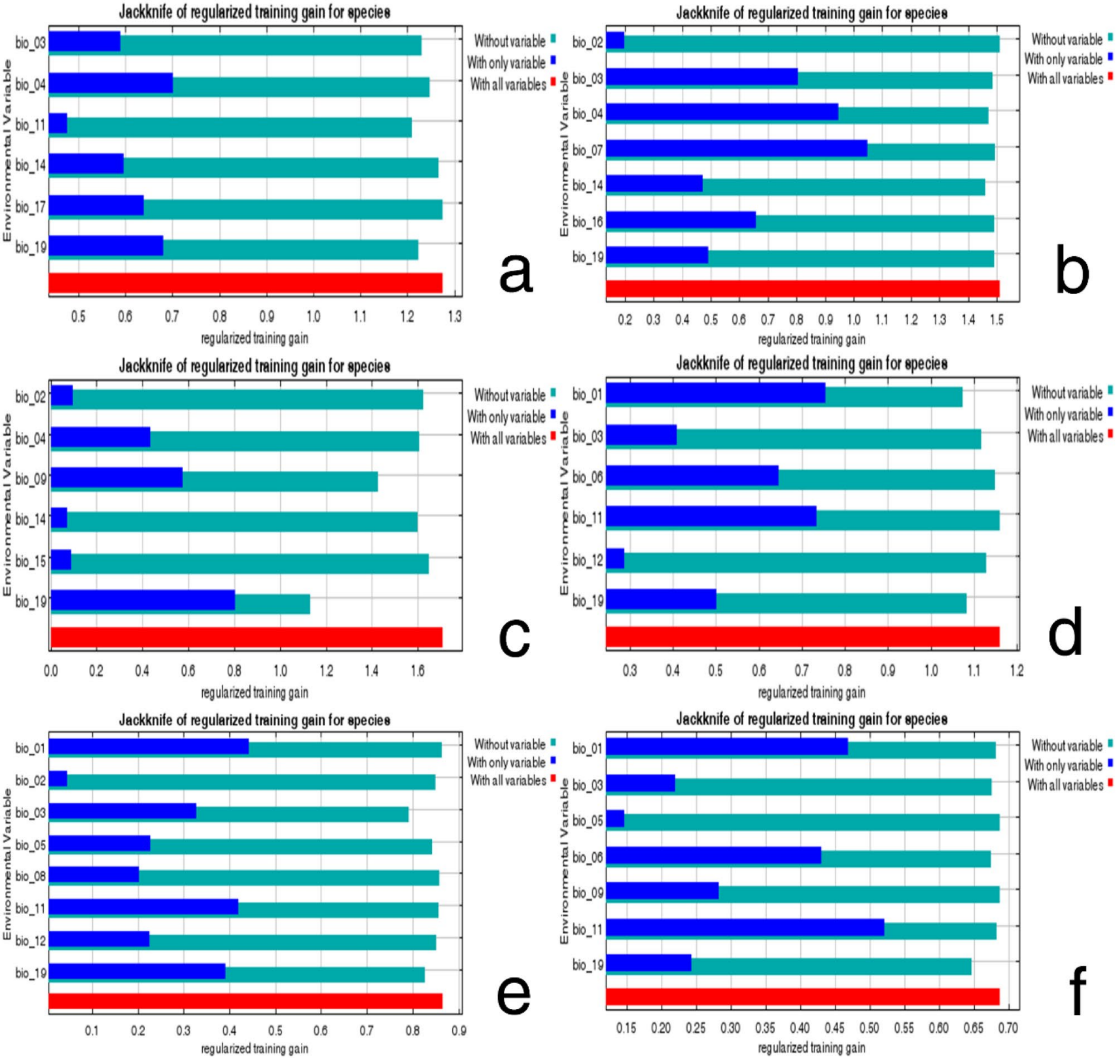
Relative importance of environmental variables of six important quarantine species based on the Jackknife test: (**a**) *Crenidorsum aroidephagus*, (**b**) *Aleurothrixus trachoides*, (**c**) *Paraleyrodes minei*, (**d**) *Nasonovia ribisnigri*, (**e**) *Macrosiphum euphorbiae*, (**f**) *Viteus vitifoliae.* Figures were created using R version 4.04 (https://www.r-project.org/).

Figure 10 and Table 1 present the environmental variables and their permutation importance, as determined in a jackknife test. In particular, precipitation of coldest quarter (Bio19), temperature seasonality (Bio04), and isothermality (Bio03) were the most influential factors on the prediction of habitat suitability for *C. aroidephagus*, and their total contribution was 74.6%. By contrast, precipitation of driest month (Bio14) had little influence on the results, with a total contribution of 3%. Bio04, Bio14 and Bio03 were the three most influential factors on the prediction of habitat suitability for *A. trachoides*, and their total contribution was 74.4%. By contrast, temperature annual range (Bio07) and Bio19 had little influence on the results, with a total contribution of 9.2%. Bio19 and mean temperature of driest quarter (Bio09) were the most influential factors on the prediction of habitat suitability for *P. minei*, and their total contribution was 73.7%. By contrast, mean diurnal range (Bio02), and precipitation seasonality (Bio15) had little influence on the results, with a total contribution of 9.5%. Bio19, mean temperature of coldest quarter (Bio11), and annual mean temperature (Bio01) were the most influential factors on the prediction of habitat suitability for *N. ribisnigri*, with a total contribution of 80.4%. By contrast, min temperature of coldest month (Bio06) and annual precipitation (Bio12) had little influence on the results, with a total contribution of 10%. Bio19, Bio03, Bio11, and Bio01 were the most influential factors on the prediction of habitat suitability for *M. euphorbiae*, with a total contribution of 83.6%. By contrast, Bio12 and mean temperature of wettest quarter (Bio08) had little influence on the results, with a total contribution of 4.5%. Bio09, Bio11, Bio19 and Bio01 were the most influential factors on the prediction of habitat suitability for *V. vitifoliae*, with a total contribution of 93.1%. By contrast, Bio09, Bio03, and max temperature of warmest month (Bio05) had little influence on the results, with a total contribution of 6.8%.

**Table 1:** Contribution rate and permutation importance of each environment variables.

		*Crenidorsum aroidephagus*		*Aleurothrixus trachoides*		*Paraleyrodes minei*		*Nasonovia ribisnigri*		*Macrosiphum euphorbiae*		*Viteus vitifoliae*	
		Percent contribution	Permutation importance	Percent contribution	Permutation importance	Percent contribution	Permutation importance	Percent contribution	Permutation importance	Percent contribution	Permutation importance	Percent contribution	Permutation importance
bio01	Annual mean temperature (°C)							20.2	37.4	12.6	0.4	18.2	31.9
bio02	Mean diurnal range (°C)			6.3	1.2	5.3	4.2			3.8	8.8		
bio03	Isothermality	15.7	14.4	14.5	47.3			9.6	5	21.5	40.3	2.5	3.9
bio04	Temperature seasonality (°C)	26.7	64.3	44.7	7.1	7.5	4.8						
bio05	Max temperature of warmest month (°C)									8.2	17.2	0.5	0
bio06	Min temperature of coldest month (°C)							6.3	15.2			21.6	26.7
bio07	Temperature annual range (°C)			4.6	24.6								
bio08	Mean temperature of wettest quarter (°C)									1	3.3		
bio09	Mean temperature of driest quarter (°C)					27.5	40.7					3.8	0
bio10	Mean temperature of warmest quarter (°C)												
bio11	Mean temperature of coldest quarter (°C)	8.4	3.7					22	15.3	15.6	8.4	24.5	30.3
bio12	Annual precipitation (mm)							3.7	7.1	3.5	4.1		
bio13	Precipitation of wettest month (mm)												
bio14	Precipitation of driest month (mm)	3	3	15.2	0.8	9.3	7.7						
bio15	Precipitation seasonality (mm)					4.2	1.7						
bio16	Precipitation of wettest quarter (mm)			10	17.9								
bio17	Precipitation of driest quarter (mm)	14.1	0										
bio18	Precipitation of warmest quarter (mm)												
bio19	Precipitation of coldest quarter (mm)	32.2	14.6	4.6	1.2	46.2	41	38.2	20.1	33.9	17.5	28.8	7.2

**Figure 11:**
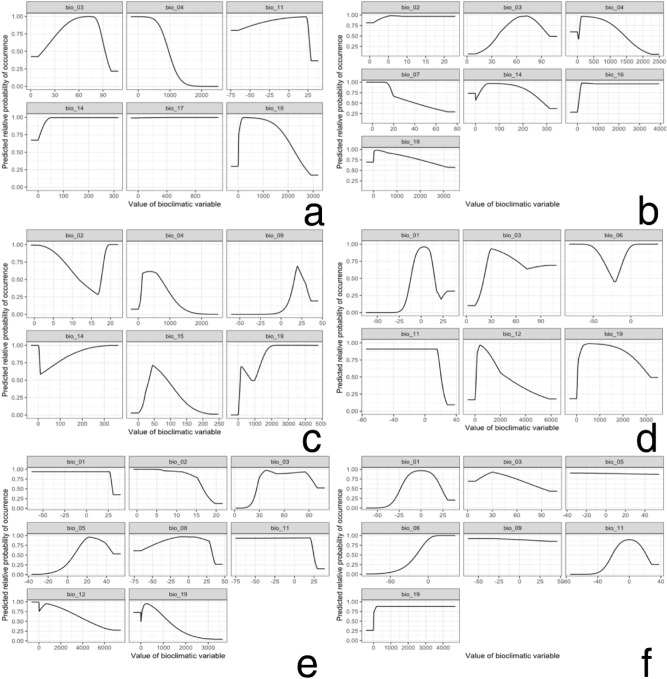
Response curves for selected variables of six important quarantine species: (**a**) *Crenidorsum aroidephagus*, (**b**) *Aleurothrixus trachoides*, (**c**) *Paraleyrodes minei*, (**d**) *Nasonovia ribisnigri*, (**e**) *Macrosiphum euphorbiae*, (**f**) *Viteus vitifoliae.* Figures were created using R version 4.04 (https://www.r-project.org/).

The response curves for the selected variables are shown in Fig. 11. These response curves indicated several patterns of variation. They were generally unimodal, monotonically increasing or decreasing, and were not overly complex or suggestive of overfitting. For example, Bio19, a key variable for *C. aroidephagus*, exhibited a unimodal pattern and Bio04 exhibited a unimodal pattern in the case of *A. trachoides*. The probability of occurrence monotonically increased with Bio19, a key factor for *P. minei.* Bio11 exhibited a unimodal pattern in the cases of *N. ribisnigri* and *M. euphorbiae*.

## Discussion

In this study, six important quarantine pests (three whitefly species and three aphid species not yet recorded in Taiwan) were selected for habitat suitability analysis prior to their invasion. The results revealed good modelling performance when potential distribution areas matched with the cultivation areas of host plants. This is the first study to successfully use SDM for the preliminary step of risk assessment of these emerging pests with limited biological data. Future work will focus on performing plausible spread simulations that take into account predicted suitability as well as potential dispersal paths. These paths depend on the existence of physical barriers such as water bodies and roads, biological constraints such as natural enemies (e.g. parasitoids, predators, and entomopathogenic fungi), and other drivers including human population density and trade nodes.

### Feasibility of SDM for evaluating potential pest invasion

The island of Taiwan covers an area of approximately 36,000 km^2^, and the highest altitude is 3952 m. Approximately 70% of the land is mountainous and hilly. Plain areas in Taiwan are mainly concentrated at the western coast. The considerable differences in terrain and altitude create a diverse ecological environment. Because of restrictions in the terrain, crops are also mainly cultivated in small areas. Therefore, if exotic phytophagous pests invade Taiwan, it is highly probable that they will find sufficient food resources and growth conditions to establish and spread the population^5^. The pest risk assessment of important international quarantine pests requires the participation of experienced insect taxonomists. However, because insect species are so diverse, conducting risk assessment based on expert experience alone can sometimes be difficult. Therefore, in this study, MaxEnt was used to predict the habitat suitability of quarantine pests, and the potential distribution areas of the host plants of pests were compared with each other.

In this study, omission rates and ROC curves were used to verify the credibility and accuracy of the habitat suitability predicted by MaxEnt^24,25^. The verification results indicated that the potential distribution areas were predicted with moderate to high accuracy (Figs. 2, 3). Jiang et al.^26^ proposed that prediction differs due to factors such as species, sample size, operation method, explanatory variables, and research scale. As the sample size (species distribution localities) increases, the accuracy of the MaxEnt prediction also increases until reaching the maximum accuracy and then eases^27^. However, large samples (such as those of 2,008 and 8,014 localities) will slightly reduce the prediction accuracy. By contrast, some models constructed with small sample sizes (< 10 localities) may also perform well. Although this study included a relatively few samples (25–136 localities), the accuracy rate was high.

A further comparison of the MaxEnt prediction areas with the crop cultivation areas revealed that except for *C. aroidephagus*, *A. trachoides*, and *N. ribisnigri* which pose low risk to crops in the their cultivation areas, the other three species pose a high risk to the host plant cultivated in a given areas (or some part thereof Figs. 4, 5, 6, 7, 8, and 9). *P. minei* poses a high risk to citrus, guava, and avocado cultivation in Taiwan, except in southwestern Taiwan; *M. euphorbiae* poses a high risk to potato, tomato and sweet potato cultivation in northern, central, and eastern Taiwan, but not in southwestern Taiwan; and *V. vitifoliae* poses a high risk to grape cultivation areas, except in southwestern Taiwan. The results of the risk assessment can also be applied to pest management. A pest monitoring mechanism can be established for areas with a high probability of invasion. In addition, our future research will aim to simulate potential spread paths as part of pest risk assessment.

### Environmental variables influencing the habitat suitability of pests

Our results revealed that the six important quarantine pests were influenced by different climatic factors (Figs. 10, 11). First, precipitations of the coldest quarter (Bio19) significantly affected habitat suitability for *C. aroidephagus*, *P. minei*, *N. ribisnigri*, *M. euphorbiae*, and *V. vitifoliae*. The response curves of Bio19 exhibited a unimodal pattern for *C. aroidephagus*, *P. minei*, *N. ribisnigri*, and *M. euphorbiae*, which suggested that the precipitations in winter limits habitat suitability for these species. By contrast, Bio19 exhibited a monotonically increasing pattern for *V. vitifoliae*, which suggests that higher precipitations in winter positively affects habitat suitability for this species. Second, seasonal temperature change is a key factor that negatively affected habitat suitability for *C. aroidephagus* and *A. trachoides*, and the results are consistent with those of previous biological studies^28–31^ and occurrence data (Fig. 1)*.* Third, the suitable temperature in driest quarter for *P. minei* was approximately 20 °C which is consistent with biological data^32^. Fourth, *N. ribisnigri* was also influenced by mean temperature of coldest quarter. Previous studies have revealed that *N. ribisnigri* grows at a temperature of 18 °C–24 °C. *M. euphorbiae* preferred areas with low temperature change, which is consistent with previous biological studies: the habitat suitability for *M. euphorbiae* decreased with temperature^33–35^. The response curve results for *P. minei* and *V. vitifoliae* are consistent with those of Zhang et al.^13^ and Liu et al.^21^.

### Uncertainties and limitations of the study based on MaxEnt

Although the study model demonstrated moderate to high accuracy, improvements are necessary to overcome the uncertainties and limitations associated with MaxEnt prediction. The first limitation of this study is the number of distribution data of species. The reliability and stability of MaxEnt increase as the sample size increases^27^. The uncertainties of distribution data are mainly due to sample size, location, background data (pseudo-absence), and spatial deviation in species sampling^36^. The error deviation of the species distribution locality will be used to select environmental variable information in the MaxEnt calculation process, resulting in the incorrect derivation of the relationship between species and environment, leading to incorrect prediction results^37–40^. Here, we applied distribution data from previous literature and global databases; thus, the accuracy of the related spatial locality could not be guaranteed. Moreover, increasing background points should be considered that does not improve model performance^12^. Next limitation was the availability and selection of environmental prediction variables, biological factors, and host plant data. Araujo and Guisan^41^ reported that predictive variables used in the prediction model should focus on the explanatory and ecological basis of environmental variables. However, in reality, predictive variables used are limited by data availability. Typically, many of the predictive variables are related to insect physiology, and the relevant variables are difficult to obtain or are lacking. For example, *C. aroidephagus* and *P. minei* analysed in this study are newly emerging pests and currently, available biological is very scarce. In addition, the default settings of MaxEnt could lead to excessive complexity and overfitting of the data; therefore, the adoption of a user-defined model is highly recommended^42^. Finally, the application of different SDMs may result in different prediction results. Many studies have compared the prediction results of different SDMs^43,44^. Zhang et al.^13^ also used MaxEnt to analyse the potential distribution areas of *V. vitifoliae* in China and compared them with those reported by Ma et al.^45^, who used CLIMEX to predict the distribution areas of *V. vitifoliae*. Although Zhang et al.^13^ pointed out the difference between the prediction results of Ma et al.^45^ and theirs, the authors believe that the prediction accuracy of CLIMEX requires a large amount of biological development data to analyse species niche. Further, certain biological development data are difficult to obtain. By contrast, MaxEnt uses the known species distribution data and environmental data as the driving force and the maximum entropy theory as the algorithm basis, which is very suitable for simulating species with limited distribution data.

## Conclusions

Invasive alien species cause major economic and ecological losses^1,2^. Therefore, prevention strategies for the invasion of alien species are considered important enough to require the attention of many countries worldwide. In fact, both whiteflies and aphids are well-known tiny agricultural pests that damage a wide range of arbor trees, vegetables and ornamental crops worldwide. They are often spread to parts of the world through trade or human activities. In this study, we selected six important quarantine pests for analysis. To our knowledge, this is the first study to predict the habitat suitability of these emerging pests. We input their current global distribution data into MaxEnt to predict the potential distribution areas of these six pests in Taiwan and compared the data with the host plant cultivation areas of the six pests to analyse their invasion risk in Taiwan. We believe that this is the first step for pest risk assessment, which can predict the potential distribution of these pests even without basic biological data. In the future, simulation of potential spread paths will be conducted for pest risk assessment.

## Methods

### Dataset of species occurrences and environmental variables

Worldwide localities of six important quarantine species were collected from CABI (https://www.cabi.org/) and Global Biodiversity Information Facility (https://www.gbif.org/), and the latitude and longitude coordinates of the location were obtained using Google Earth Pro. The occurrence of repeated data due to the different sources of the collected distribution data may cause research bias and have other influences, leading to increased density of the species distribution data in a certain area; this may lead to deviations in the prediction results^10,11,21^. To avoid affecting the prediction results, we excluded duplicate and unclear distribution localities by using Quantum GIS (QGIS)^46^ and ensuring the presence of only one distribution point in each raster. In total, 25 localities of *C. aroidephagus*, 62 localities of *A. trachoides*, 44 localities of *P. minei*, 68 localities of *N. ribisnigri*, 136 localities of *M. euphorbiae*, and 99 localities of *V. vitifoliae* were included for analysis (Fig. 1, Supplementary Tables S1–S6). All distribution data were organised using Microsoft Excel and in accordance with the MaxEnt software format requirements and were saved in .csv file format for further analysis^47^.

We used a set of 19 environmental variables from WorldClim v.2.1 (https://www.worldclim.org)^49^ with the ‘current period’ of 1970–2000 at a spatial resolution of 30 arc-seconds (1 km^2^). These data contain 19 variables of these years derived from monthly temperature and precipitation values, representing annual trends, seasonality, and extreme or restrictive environmental factors. This bioclimatic variable factor is related to the distribution and survival of small arthropods^49^ and has been widely used in global studies of species habitat suitability prediction^50^. We extracted 19 bioclimatic variables from the WorldClim database by R studio to use the R packages ‘raster’^51^ and ‘rgdal’^52^. These variables were imported into MaxEnt for initial model to calculate the contribution rate using jackknife test to avoid multicollinearity. The selected variables are shown in Table 1.

### Distribution modelling

MaxEnt v.3.1.4^53^ was used to predict the habitat suitability of the aforementioned six important quarantine pests in Taiwan (i.e., if they are introduced) and outlying islands on the basis of their global distribution data (Fig. 1, Supplementary Table S1–S6) and environmental variables (Table 1) through the use of the R package ‘dismo’^54^. The R package ‘ENMeval’^55^ was applied to evaluate the performance of various models and parameters in relation to the best fitting model in ten-fold replicates. The logistic output was chosen as an estimate of the probability of the outcome being present conditional on the variables, per grid cell. This study only used presence data to generate pseudo-absences, and 10,000 background points were randomly selected by the MaxEnt model. The MaxEnt model ran either 500 iterations of these processes or continued until a convergence threshold of 0.00001 was met. Parameters were set based on best models for each species; the five feature types (i.e., linear, quadratic, product, threshold, and hinge) were allowed in the modelling and the regularisation multiplier was set to 0.5. A jackknife test was conducted to screen for dominant climatic factors and to calculate the contribution of various bioclimatic variables to pest habitat suitability (Fig. 10)^56^. In addition, response curves for selected variables were also generated (Fig. 11).

The prediction results from Maxent modelling were evaluated according to threshold-independent AUC values, calculated in R software. ROC curves were used to plot the true-positive rate against the false-positive rate, and the AUC was used to assess the predictive accuracy of models^8,24^. We selected a test sensitivity of 0% and an omission rate (OR) of 10%^57,58^. The AUC value ranged from 0 to 1, where higher values indicate higher predictive accuracy of models^22,23^. In the case of the default rate (OR), the value at 10% was 0.10, and the sensitivity test value at 0% was 0; poor performance is indicated by a value exceeding the predicted rate^59^. For each species, the final models were generated for probable distributions in Taiwan (Figs. 4, 5, 6, 7, 8, and 9) and the globe (Supplementary Fig. S1).

### Host crop mapping

We mainly selected economically important host crops for these six important quarantine pests. One host plant was chosen for oligophagous pests, *A. andraeanum* for *C. aroidephagus*^31^, *L. sativa* for *N. ribisnigri*^60^, and *Vitis vinifera* for *V. vitifoliae*^61^. For polyphagous pests, three crops were selected: tomato, sweet potato, and potato for *A. trachoides* and *M. euphorbia*^30,62^. The hosts of *P. minei* are citrus, avocado, and guava^63^. We then obtained data on the 2019 Taiwan crop cultivation areas listed by the Agriculture and Food Administration of the Executive Yuan from the agricultural report resource network database by using towns (or district) as a point. Subsequently, we obtained the coordinates of the locations using Google Earth Pro and located the crop areas using QGIS^46^. The crop cultivation maps were compared with the prediction results of MaxEnt (Figs. 4, 5, 6, 7, 8, and 9) and a histogram of the habitat suitability of crop locations for six important quarantine species was generated (Supplementary Fig. S2).

## Supplementary Information

Supplementary Information.
