# Supplementary material for: Assessment of potential invasion for six phytophagous quarantine pests in Taiwan
Source: Sci Rep. 2021 May 21;11:10666. doi: 10.1038/s41598-021-89914-w (PMC8140104; doi:10.1038/s41598-021-89914-w)
Supplement: Supplementary file 1 — Supplementary Information. [file 41598_2021_89914_MOESM1_ESM.pdf]

# **Assessment of potential invasion for six phytophagous quarantine pests in Taiwan**

Hsin-Ting Yeh[1], Harn-Yeu Cheah[24], Ming-Chih Chiu[3*], Jhih-Rong Liao[4*], and Chiun-Cheng Ko[4†]

[1] The Experimental Forest, College of Bio-Resources and Agriculture, National Taiwan University, Zhushan Township, Nantou County 557004, Taiwan

[2] Master Program for Plant Medicine, College of Bio-Resources and Agriculture, National Taiwan University, Taipei City 106332, Taiwan

[3] Center for Marine Environmental Studies (CMES), Ehime University, Matsuyama, Ehime 7908577, Japan

[4] Department of Entomology, National Taiwan University, Taipei City 106332, Taiwan

[*] **Co-corresponding authors:** Ming-Chih Chiu: mingchih.chiu@gmail.com

Jhih-Rong Liao: k1107053@hotmail.com

[†]This paper is dedicated to the memory of the late Chiun-Cheng Ko.

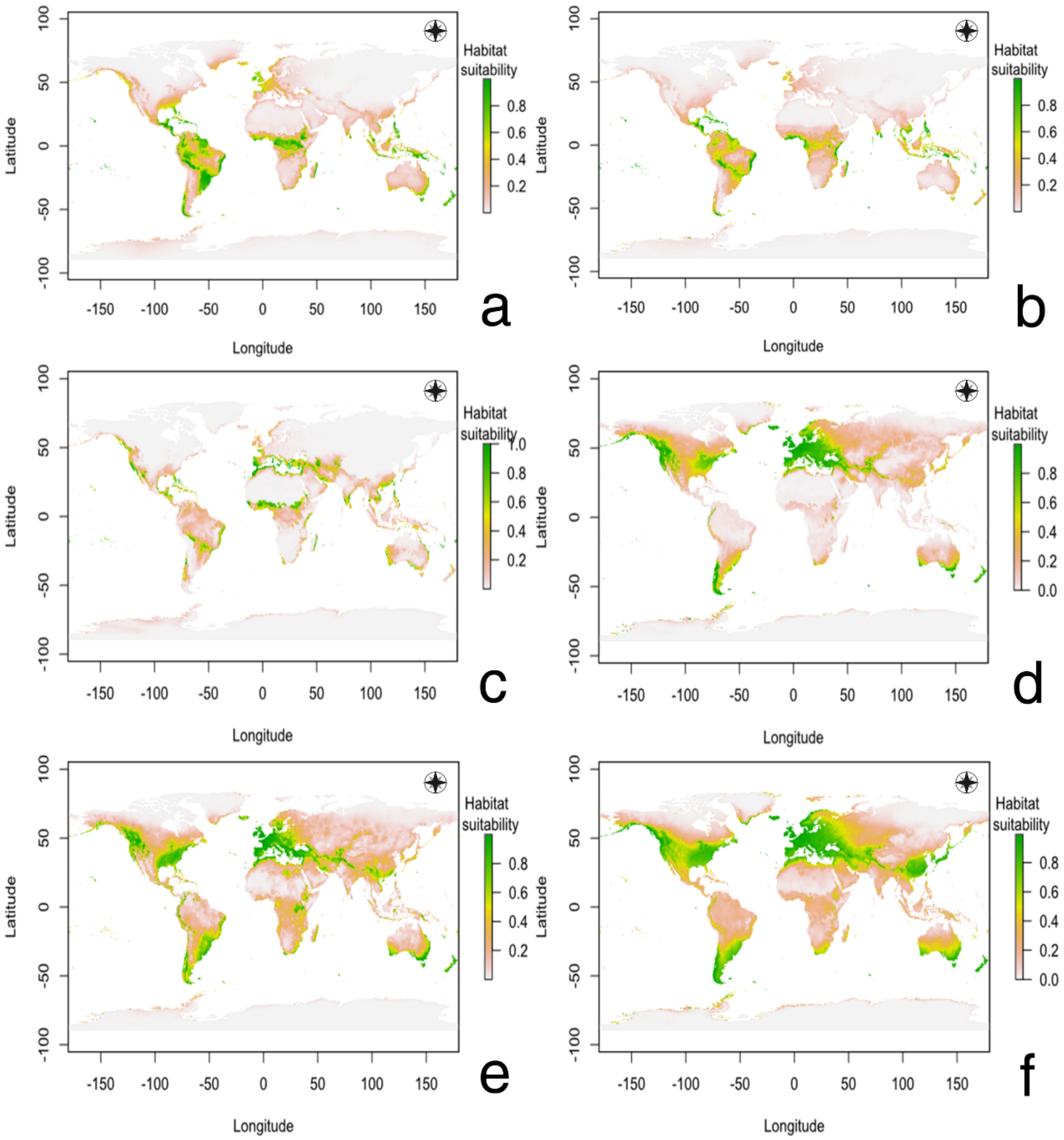


Figure S1. Global potential distribution of six important quarantine species: (a) *Crenidorsum aroidephagus*, (b) *Aleurothrixus trachoides*, (c) *Paraleyrodes minei*, (d) *Nasonovia ribisnigri*, (e) *Macrosiphum euphorbiae*, (f) *Viteus vitifoliae*. Maps were created using R version 4.04 (https://www.r-project.org/).

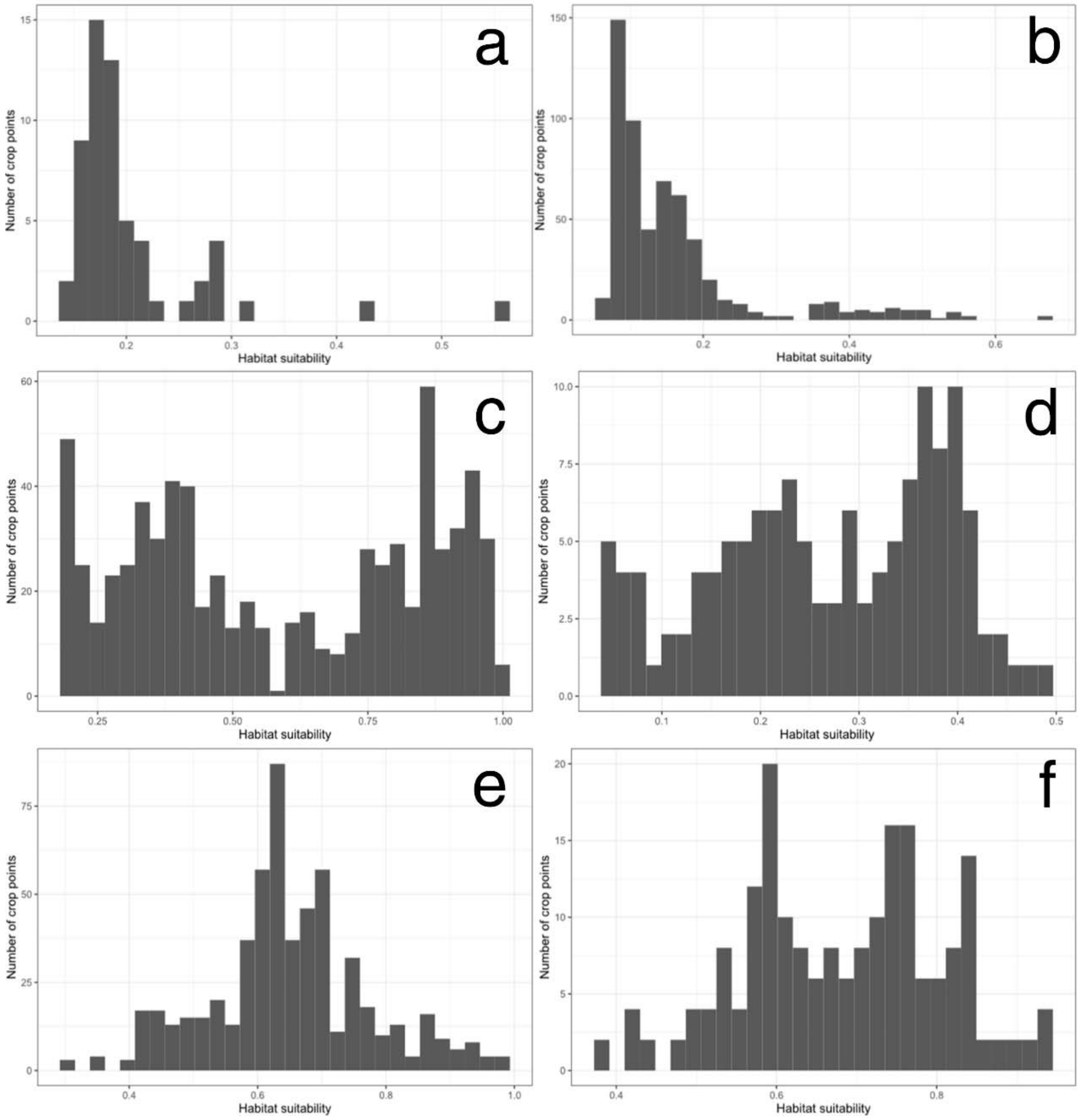


Figure S2. Proportion of vulnerable crop locations with habitat suitability of six important quarantine species: (a) *Crenidorsum aroidephagus*, (b) *Aleurothrixus trachoides*, (c) *Paraleyrodes minei*, (d) *Nasonovia ribisnigri*, (e) *Macrosiphum euphorbiae*, (f) *Viteus vitifoliae*. Figures were created using R version 4.04 (https://www.r-project.org/).

Supplementary Table S1. Occurrence data of *Crenidorsum aroidephagus*

| | **Latitude** | **Longitude** | **Location** | **Nation** | **Reference** |
|---|---|---|---|---|---|
| *Crenidorsum aroidephagus* | 21.533554 | -100.842996 | | Mexico | Martin *et al* ., 2001 |
| *Crenidorsum aroidephagus* | 36.778251 | -119.417939 | California | | Martin *et al* ., 2001 |
| *Crenidorsum aroidephagus* | 27.664825 | -81.515752 | Florida | USA | Martin *et al* ., 2001 |
| *Crenidorsum aroidephagus* | 19.896766 | -155.582784 | Hawaii | | Martin *et al* ., 2001 |
| *Crenidorsum aroidephagus* | 17.189877 | -88.49765 | | Belize | Martin *et al* ., 2001 |
| *Crenidorsum aroidephagus* | 9.748917 | -83.753428 | | Costa Rica | Martin *et al* ., 2001 |
| *Crenidorsum aroidephagus* | 15.209193 | -90.368821 | | Guatemala | Martin *et al* ., 2001 |
| *Crenidorsum aroidephagus* | 8.537982 | -80.782127 | | Panama | Martin *et al* ., 2001 |
| *Crenidorsum aroidephagus* | -14.23504 | -51.925275 | | Brazil | Martin *et al* ., 2001 |
| *Crenidorsum aroidephagus* | 4.570868 | -74.297334 | | Columbia | Martin *et al* ., 2001 |
| *Crenidorsum aroidephagus* | 6.42375 | -66.589729 | | Venezuela | Martin *et al* ., 2001 |
| *Crenidorsum aroidephagus* | 13.193887 | -59.543198 | | Barbados | Martin *et al* ., 2001 |
| *Crenidorsum aroidephagus* | 21.521759 | -77.781166 | | Cuba | Martin *et al* ., 2001 |
| *Crenidorsum aroidephagus* | 15.414999 | -61.370977 | | Dominica | Martin *et al* ., 2001 |
| *Crenidorsum aroidephagus* | 18.735694 | -70.162652 | | Dominican Republic | Martin *et al* ., 2001 |
| *Crenidorsum aroidephagus* | 18.220833 | -66.590148 | | Puerto Rico | Martin *et al* ., 2001 |
| *Crenidorsum aroidephagus* | 10.691803 | -61.222503 | | Trinidad and Tobago | Martin *et al* ., 2001 |
| *Crenidorsum aroidephagus* | 46.227639 | 2.21375 | | France | CABI, 2019 |
| *Crenidorsum aroidephagus* | 51.165688 | 10.451525 | | Germany | CABI, 2019 |
| *Crenidorsum aroidephagus* | 39.399876 | -8.224448 | | Portugal | CABI, 2019 |
| *Crenidorsum aroidephagus* | 20.593678 | 78.96288 | | India | Martin *et al* ., 2001 |
| *Crenidorsum aroidephagus* | -17.713372 | 178.065032 | | Fiji | Martin *et al* ., 2001 |
| *Crenidorsum aroidephagus* | 12.879731 | 121.774023 | | Philippines | Martin *et al* ., 2001 |
| *Crenidorsum aroidephagus* | -14.26 | -170.75 | | American Samoa | Martin *et al* ., 2001 |
| *Crenidorsum aroidephagus* | 4.210484 | 101.975766 | | Malaysia | GBIF |

Supplementary Table S2. Occurrence data of *Aleurothrixus trachoides*

| | Latitude | Longitude | Location | Nation | Reference |
|---|---|---|---|---|---|
| *Aleurothrixus trachoides* | -9.274053 | 142.223094 | 3oigu Island, Queenslanc | Australia | siness Queensland-Capsium whitefly |
| *Aleurothrixus trachoides* | -11.6455 | 43.3333 | | Comoros | EPPO, 2018 |
| *Aleurothrixus trachoides* | 13.443182 | -15.310139 | | Gambia | EPPO, 2018 |
| *Aleurothrixus trachoides* | -12.8275 | 45.166244 | | Mayotte | EPPO, 2018 |
| *Aleurothrixus trachoides* | -18.665695 | 35.529557 | | Mozambique | EPPO, 2018 |
| *Aleurothrixus trachoides* | 9.081999 | 8.675277 | | Nigeria | EPPO, 2018 |
| *Aleurothrixus trachoides* | -21.115141 | 55.536384 | | Reunion | EPPO, 2018 |
| *Aleurothrixus trachoides* | -6.369027 | 34.888821 | | Tanzania | EPPO, 2018 |
| *Aleurothrixus trachoides* | 17.060816 | -61.796428 | | Antigua and Barbuda | EPPO, 2018 |
| *Aleurothrixus trachoides* | 25.03428 | -77.396276 | | Bahamas | EPPO, 2018 |
| *Aleurothrixus trachoides* | 13.193887 | -59.543198 | | Barbados | EPPO, 2018 |
| *Aleurothrixus trachoides* | 17.189877 | -88.49765 | | Belize | EPPO, 2018 |
| *Aleurothrixus trachoides* | -12.579738 | -41.700727 | Bahia | Brazil | EPPO, 2018 |
| *Aleurothrixus trachoides* | -22.906848 | -43.172897 | Rio de Janeiro | | |
| *Aleurothrixus trachoides* | 19.3133 | -81.2546 | | Cayman Islands | EPPO, 2018 |
| *Aleurothrixus trachoides* | 4.570868 | -74.297331 | | Colombia | EPPO, 2018 |
| *Aleurothrixus trachoides* | 9.748917 | -83.753428 | | Costa Rica | EPPO, 2018 |
| *Aleurothrixus trachoides* | 21.521756 | -77.781167 | | Cuba | EPPO, 2018 |
| *Aleurothrixus trachoides* | 12.16957 | -68.99002 | | Curaçao | Kumar *et al* ., 2016 |
| *Aleurothrixus trachoides* | 15.414999 | -61.370976 | | Dominica | EPPO, 2018 |
| *Aleurothrixus trachoides* | 18.735693 | -70.162651 | | Dominican Republic | EPPO, 2018 |
| *Aleurothrixus trachoides* | -0.668438 | -90.694468 | Galapagos | Ecuador | EPPO, 2018 |
| *Aleurothrixus trachoides* | 13.794185 | -88.89653 | | El Salvador | EPPO, 2018 |
| *Aleurothrixus trachoides* | 3.933889 | -53.125782 | | French Guiana | EPPO, 2018 |
| *Aleurothrixus trachoides* | 16.265 | -61.551 | | Guadeloupe | EPPO, 2018 |
| *Aleurothrixus trachoides* | 15.783471 | -90.230759 | | Guatemala | EPPO, 2018 |
| *Aleurothrixus trachoides* | 4.860417 | -58.93018 | | Guyana | EPPO, 2018 |
| *Aleurothrixus trachoides* | 18.971187 | -72.285215 | | Haiti | EPPO, 2018 |
| *Aleurothrixus trachoides* | 15.199999 | -86.241905 | | Honduras | EPPO, 2018 |

| | | | | | |
|---|---|---|---|---|---|
| *Aleurothrixus trachoides* | 18.109581 | -77.297508 | | Jamaica | EPPO, 2018 |
| *Aleurothrixus trachoides* | 14.641528 | -61.024174 | | Martinique | EPPO, 2018 |
| *Aleurothrixus trachoides* | 23.634502 | -102.552786 | | Mexico | EPPO, 2018 |
| *Aleurothrixus trachoides* | 52.376356 | 4.924567 | | Netherlands Antilles | EPPO, 2018 |
| *Aleurothrixus trachoides* | 12.865416 | -85.207229 | | Nicaragua | EPPO, 2018 |
| *Aleurothrixus trachoides* | 8.537981 | -80.782127 | | Panama | EPPO, 2018 |
| *Aleurothrixus trachoides* | -6.314993 | 143.955551 | | Papua New Guinea | EPPO, 2018 |
| *Aleurothrixus trachoides* | -9.189966 | -75.015152 | | Peru | EPPO, 2018 |
| *Aleurothrixus trachoides* | 18.220833 | -66.590149 | | Puerto Rico | EPPO, 2018 |
| *Aleurothrixus trachoides* | -16.761301 | -151.443589 | | Society Islands | Mound and Halsey , 1978 |
| *Aleurothrixus trachoides* | -9.645712 | 160.156194 | | Solomon Islands | Kumar *et al* ., 2016 |
| *Aleurothrixus trachoides* | 3.919305 | -56.027782 | | Suriname | EPPO, 2018 |
| *Aleurothrixus trachoides* | 10.691803 | -61.222503 | | Trinidad and Tobago | EPPO, 2018 |
| *Aleurothrixus trachoides* | 21.694025 | -71.797928 | | Turks and Caicos Islands | EPPO, 2018 |
| *Aleurothrixus trachoides* | 36.778263 | -119.417933 | California | | |
| *Aleurothrixus trachoides* | 27.664827 | -81.515753 | Florida | | |
| *Aleurothrixus trachoides* | 19.896757 | -155.582773 | Hawaii | USA | EPPO, 2018 |
| *Aleurothrixus trachoides* | 30.984297 | -91.962333 | Louisiana | | |
| *Aleurothrixus trachoides* | 31.968599 | -99.901813 | Texas | | |
| *Aleurothrixus trachoides* | 6.42375 | -66.589731 | | Venezuela | EPPO, 2018 |
| *Aleurothrixus trachoides* | 18.42309 | -64.618935 | | Virgin Islands (British) | EPPO, 2018 |
| *Aleurothrixus trachoides* | 18.333333 | -64.75 | | Virgin Islands (US) | EPPO, 2018 |
| *Aleurothrixus trachoides* | 15.317277 | 75.713889 | Karnataka | | EPPO, 2018 |
| *Aleurothrixus trachoides* | 10.850515 | 76.271083 | Kerala | India | |
| *Aleurothrixus trachoides* | 19.75148 | 75.713888 | Maharashtra | | Sundararaj *et al* ., 2018 |
| *Aleurothrixus trachoides* | 11.127122 | 78.656894 | Tamil Nadu | | |
| *Aleurothrixus trachoides* | 55.378051 | -3.435974 | | United Kingdom | EPPO, 2018 |
| *Aleurothrixus trachoides* | -17.713371 | 178.065032 | | Fiji | EPPO, 2018 |
| *Aleurothrixus trachoides* | -17.638665 | -149.406835 | | French Polynesia | EPPO, 2018 |
| *Aleurothrixus trachoides* | 13.444304 | 144.793731 | | Guam | EPPO, 2018 |
| *Aleurothrixus trachoides* | 7.425553 | 150.55082 | | Micronesia | EPPO, 2018 |

| | | | | |
|---|---|---|---|---|
| *Aleurothrixus trachoides* | -0.522778 | 166.931503 | Nauru | EPPO, 2018 |
| *Aleurothrixus trachoides* | -21.178985 | -175.198241 | Tonga | EPPO, 2018 |

Supplementary Table S3. Occurrence data of *Paraleyrodes minei*

| | Latitude | Longitude | Location | Nation | Reference |
|---|---|---|---|---|---|
| *Paraleyrodes minei* | 9.30769 | 2.315834 | | Benin | EPPO, 2019 |
| *Paraleyrodes minei* | 7.946527 | -1.023194 | | Ghana | EPPO, 2019 |
| *Paraleyrodes minei* | 31.791702 | -7.092619 | | Morocco | EPPO, 2019 |
| *Paraleyrodes minei* | 17.189877 | -88.49765 | | Belize | EPPO, 2019 |
| *Paraleyrodes minei* | 32.3078 | -64.7505 | | Bermuda | EPPO, 2019 |
| *Paraleyrodes minei* | 4.570868 | -74.297331 | | Colombia | EPPO, 2019 |
| *Paraleyrodes minei* | 18.735694 | -70.162652 | | Dominican Republic | EPPO, 2019 |
| *Paraleyrodes minei* | 15.783471 | -90.230759 | | Guatemala | EPPO, 2019 |
| *Paraleyrodes minei* | 18.971187 | -72.285215 | | Haiti | EPPO, 2019 |
| *Paraleyrodes minei* | 15.199999 | -86.241905 | | Honduras | EPPO, 2019 |
| *Paraleyrodes minei* | 23.634501 | -102.552784 | | Mexico | EPPO, 2019 |
| *Paraleyrodes minei* | 18.220833 | -66.590149 | | Puerto Rico | EPPO, 2019 |
| *Paraleyrodes minei* | 36.778263 | -119.417933 | California | USA | EPPO, 2019 |
| *Paraleyrodes minei* | 27.664827 | -81.515753 | Florida | | |
| *Paraleyrodes minei* | 19.896757 | -155.582773 | Hawaii | | |
| *Paraleyrodes minei* | 31.968599 | -99.901813 | Texas | | |
| *Paraleyrodes minei* | 32.715738 | -117.161084 | San Diego | | Bellows *et al* ., 1998 |
| *Paraleyrodes minei* | 19.566395 | 109.949686 | Hainan | China | EPPO, 2019 |
| *Paraleyrodes minei* | 22.319304 | 114.169361 | | Hong Kong | EPPO, 2019 |
| *Paraleyrodes minei* | 32.427901 | 53.688042 | | Iran | EPPO, 2019 |
| *Paraleyrodes minei* | 31.04605 | 34.851611 | | Israel | EPPO, 2019 |
| *Paraleyrodes minei* | 10.850516 | 76.271084 | Kerala | India | Poorani, 2019 |
| *Paraleyrodes minei* | 12.843781 | 75.247906 | Dakshina Kannada | | Sujithra *et al* ., 2019 |
| *Paraleyrodes minei* | 12.469133 | 75.094078 | Kasaragod | | |
| *Paraleyrodes minei* | 15.317277 | 75.713889 | Karnataka | | |
| *Paraleyrodes minei* | 13.340881 | 74.742143 | Udupi | | |
| *Paraleyrodes minei* | 11.740086 | 92.65864 | Andaman and Nicobar Islands | | Dubey, 2019 |
| *Paraleyrodes minei* | 33.854721 | 35.862285 | | Lebanon | EPPO, 2019 |
| *Paraleyrodes minei* | 4.210484 | 101.975766 | | Malaysia | EPPO, 2019 |

| | | | | | |
|---|---|---|---|---|---|
| *Paraleyrodes minei* | 1.352083 | 103.819836 | | Singapore | EPPO, 2019 |
| *Paraleyrodes minei* | 34.802075 | 38.996815 | | Syria | EPPO, 2019 |
| *Paraleyrodes minei* | 35.126413 | 33.429859 | | Cyprus | EPPO, 2019 |
| *Paraleyrodes minei* | 35.240117 | 24.809269 | Kriti | Greece | EPPO, 2019 |
| *Paraleyrodes minei* | 37.599994 | 14.015356 | Sicilia | | EPPO, 2019 |
| *Paraleyrodes minei* | 37.507877 | 15.08303 | Catania | | |
| *Paraleyrodes minei* | 38.193733 | 15.554206 | Messina | Italy | Longo and Rapisarda, 2014 |
| *Paraleyrodes minei* | 37.075474 | 15.286586 | Siracusa | | |
| *Paraleyrodes minei* | 40.814122 | 14.339067 | Portici | | Iaccarino *et al* ., 2011 |
| *Paraleyrodes minei* | 35.937496 | 14.375416 | | Malta | EPPO, 2019 |
| *Paraleyrodes minei* | 37.741248 | -25.675596 | Azores | Portugal | EPPO, 2019 |
| *Paraleyrodes minei* | 32.760707 | -16.959472 | Madeira | | |
| *Paraleyrodes minei* | 28.291565 | -16.629128 | Canary Island | Spain | EPPO, 2019 |
| *Paraleyrodes minei* | 36.716746 | -4.425996 | Malaga | | Iaccarino *et al* ., 2011 |
| *Paraleyrodes minei* | 38.963745 | 35.243322 | | Turkey | EPPO, 2019 |

Supplementary Table S4. Occurrence data of *Nasonovia ribisnigri*

| | Latitude | Longitude | Location | Nation | Reference |
|---|---|---|---|---|---|
| *Nasonovia ribisnigri* | 47.516229 | 14.550071 | | Austria | CABI, 2003 |
| *Nasonovia ribisnigri* | 50.503887 | 4.469936 | | Belgium | CABI, 2003 |
| *Nasonovia ribisnigri* | 42.733884 | 25.48583 | | Bulgaria | CABI, 2003 |
| *Nasonovia ribisnigri* | 49.817492 | 15.472962 | | Czech Republic | CABI, 2003 |
| *Nasonovia ribisnigri* | 56.26392 | 9.501785 | | Denmark | CABI, 2003 |
| *Nasonovia ribisnigri* | 61.892635 | -6.911806 | | Faroe Islands | CABI, 2003 |
| *Nasonovia ribisnigri* | 61.924111 | 25.748152 | | Finland | CABI, 2003 |
| *Nasonovia ribisnigri* | 46.227638 | 2.213749 | | France | CABI, 2003 |
| *Nasonovia ribisnigri* | 51.165692 | 10.451526 | | Germany | CABI, 2003 |
| *Nasonovia ribisnigri* | 39.074207 | 21.824312 | | Greece | CABI, 2003 |
| *Nasonovia ribisnigri* | 47.162493 | 19.503305 | | Hungary | CABI, 2003 |
| *Nasonovia ribisnigri* | 53.277639 | -7.967972 | | Ireland | CABI, 2003 |
| *Nasonovia ribisnigri* | 40.120875 | 9.012893 | Sardinia | Italy | Luciano et al., 1989 |
| *Nasonovia ribisnigri* | 56.879635 | 24.603189 | | Latvia | CABI, 2003 |
| *Nasonovia ribisnigri* | 55.169437 | 23.881275 | | Lithuania | CABI, 2003 |
| *Nasonovia ribisnigri* | 47.411629 | 28.369884 | | Moldova | CABI, 2003 |
| *Nasonovia ribisnigri* | 52.132633 | 5.291266 | | Netherlands | CABI, 2003 |
| *Nasonovia ribisnigri* | 60.472024 | 8.468944 | | Norway | CABI, 2003 |
| *Nasonovia ribisnigri* | 51.919439 | 19.145136 | | Poland | CABI, 2003 |
| *Nasonovia ribisnigri* | 39.399871 | -8.224452 | | Portugal | CABI, 2003 |
| *Nasonovia ribisnigri* | 32.760707 | -16.959473 | | Madeira | CABI, 2003 |
| *Nasonovia ribisnigri* | 45.943161 | 24.966762 | | Romania | CABI, 2003 |
| *Nasonovia ribisnigri* | 61.524005 | 105.318756 | | Russia | CABI, 2003 |
| *Nasonovia ribisnigri* | 48.669027 | 19.699025 | | Slovakia | CABI, 2003 |
| *Nasonovia ribisnigri* | 40.313341 | -3.478635 | La Poveda Experimental Farm | Spain | Morales et al., 2013 |
| *Nasonovia ribisnigri* | 28.291565 | -16.629128 | | Canary Islands | CABI, 2003 |
| *Nasonovia ribisnigri* | 60.128161 | 18.643499 | | Sweden | CABI, 2003 |
| *Nasonovia ribisnigri* | 46.818188 | 8.227512 | | Switzerland | CABI, 2003 |
| *Nasonovia ribisnigri* | 55.378502 | -3.435974 | | UK/Britain | CABI, 2003 |

| | | | | | |
|---|---|---|---|---|---|
| *Nasonovia ribisnigri* | 48.379432 | 31.165582 | | Ukraine | CABI, 2003 |
| *Nasonovia ribisnigri* | 40.069129 | 45.050793 | | Armenia | CABI, 2003 |
| *Nasonovia ribisnigri* | 40.143104 | 47.576928 | | Azerbaijan | CABI, 2003 |
| *Nasonovia ribisnigri* | 42.315408 | 43.356891 | | Republic of Georgia | CABI, 2003 |
| *Nasonovia ribisnigri* | 48.019571 | 66.923686 | | Kazakhstan | CABI, 2003 |
| *Nasonovia ribisnigri* | 33.854721 | 35.862285 | | Lebanon | CABI, 2003 |
| *Nasonovia ribisnigri* | 38.963745 | 35.243323 | | Turkey | CABI, 2003 |
| *Nasonovia ribisnigri* | 58.8 | -108.02 | | Canada | CABI, 2003 |
| *Nasonovia ribisnigri* | 49.37641 | -121.815931 | fraser valley | British Columbia | CABI, 2003 |
| *Nasonovia ribisnigri* | 49.111534 | -122.734943 | Cloverdale | | orbes and Mackenzie, 1982 |
| *Nasonovia ribisnigri* | 46.565316 | -66.461917 | | New Brunswick | CABI, 2003 |
| *Nasonovia ribisnigri* | 51.253776 | -85.323214 | | Ontario | CABI, 2003 |
| *Nasonovia ribisnigri* | 52.939916 | -73.549138 | | Quebec | CABI, 2003 |
| *Nasonovia ribisnigri* | 34.048928 | -111.093731 | Arizona | USA | Palumbo, 2000 |
| *Nasonovia ribisnigri* | 36.677737 | -121.655501 | salinas valley,California | | Hopper et al., 2011 |
| *Nasonovia ribisnigri* | 44.068199 | -114.742039 | Idaho | | CABI, 2003 |
| *Nasonovia ribisnigri* | 42.407211 | -71.382437 | Massachusetts | | CABI, 2003 |
| *Nasonovia ribisnigri* | 46.879681 | -110.362567 | Montana | | CABI, 2003 |
| *Nasonovia ribisnigri* | 40.712776 | -74.005973 | New York | | CABI, 2003 |
| *Nasonovia ribisnigri* | 43.804133 | -120.554202 | Oregon | | CABI, 2003 |
| *Nasonovia ribisnigri* | 41.203322 | -77.194525 | Pennsylvania | | CABI, 2003 |
| *Nasonovia ribisnigri* | 44.558803 | -72.577842 | Vermont | | CABI, 2003 |
| *Nasonovia ribisnigri* | 47.751071 | -120.740137 | Washington | | CABI, 2003 |
| *Nasonovia ribisnigri* | -39.313556 | -63.396171 | | Argentina | CABI, 2003 |
| *Nasonovia ribisnigri* | -30.034627 | -51.2177 | Rio Grande do Sul | Brazil | CABI, 2003 |
| *Nasonovia ribisnigri* | -23.55052 | -46.633309 | Sao Paulo | | CABI, 2003 |
| *Nasonovia ribisnigri* | -35.67516 | -71.542976 | | Chile | CABI, 2003 |
| *Nasonovia ribisnigri* | -9.189969 | -75.015152 | | Peru | CABI, 2003 |
| *Nasonovia ribisnigri* | -43.754228 | 171.163724 | Canterbury | New Zealand | Fagan et al., 2010 |
| *Nasonovia ribisnigri* | 31.318327 | 48.670619 | Ahvaz | Iran | Nazari et al., 2012 |
| *Nasonovia ribisnigri* | -9.166667 | -77.75 | Callejón de Huaylas zone | Perú | Mallqui et al., 2011 |

| | | | | | |
|---|---|---|---|---|---|
| *Nasonovia ribisnigri* | 40.48301 | -4.087557 | | Iberian Peninsula | Díaz et al., 2008 |
| *Nasonovia ribisnigri* | 45.512654 | 4.490451 | Southern | France | Rufingier et al., 1999 |
| *Nasonovia ribisnigri* | -31.253218 | 146.9211 | New South Wales | | |
| *Nasonovia ribisnigri* | -20.917576 | 142.702792 | Queensland | | |
| *Nasonovia ribisnigri* | -30.000227 | 136.209157 | South Australia | Australia | Plant Heat Australia |
| *Nasonovia ribisnigri* | -41.45452 | 145.970665 | Tasmania | | |
| *Nasonovia ribisnigri* | -37.471306 | 144.785154 | Victoria | | |
| *Nasonovia ribisnigri* | -27.67282 | 121.628307 | Western Australia | | |

Supplementary Table S5. Occurrence data of *Macrosiphum euphorbiae*

| | Latitude | Longitude | Location | Nation | Reference |
|---|---|---|---|---|---|
| *Macrosiphum euphorbiae* | 35.563419 | 6.189 | Batna | | |
| *Macrosiphum euphorbiae* | 35.627419 | 6.373399 | El Madher | Algeria | Laamari et al., 2010(2009) |
| *Macrosiphum euphorbiae* | 36.034955 | 5.338323 | Guellal | | |
| *Macrosiphum euphorbiae* | -10.722793 | 18.691479 | | Angola | CABI, 2019 |
| *Macrosiphum euphorbiae* | -3.373056 | 29.918886 | | Burundi | CABI, 2019 |
| *Macrosiphum euphorbiae* | -4.038333 | 21.758663 | | Congo | CABI, 2019 |
| *Macrosiphum euphorbiae* | 26.820554 | 30.802496 | | Egypt | CABI, 2019 |
| *Macrosiphum euphorbiae* | 9.145 | 40.489673 | | Ethiopia | CABI, 2019 |
| *Macrosiphum euphorbiae* | -0.023559 | 37.906193 | | Kenya | CABI, 2019 |
| *Macrosiphum euphorbiae* | 26.335101 | 17.228329 | | Libya | CABI, 2019 |
| *Macrosiphum euphorbiae* | -13.254307 | 34.301525 | | Malawi | CABI, 2019 |
| *Macrosiphum euphorbiae* | -20.348404 | 57.552153 | | Mauritius | CABI, 2019 |
| *Macrosiphum euphorbiae* | 31.791701 | -7.09262 | | Morocco | CABI, 2019 |
| *Macrosiphum euphorbiae* | -18.665683 | 35.529554 | | Mozambique | CABI, 2019 |
| *Macrosiphum euphorbiae* | -21.115141 | 55.536384 | | Réunion | CABI, 2019 |
| *Macrosiphum euphorbiae* | -1.940278 | 29.873888 | | Rwanda | CABI, 2019 |
| *Macrosiphum euphorbiae* | -15.96501 | -5.708924 | | Saint Helena | CABI, 2019 |
| *Macrosiphum euphorbiae* | -6.369028 | 34.888823 | | Tanzania | CABI, 2019 |
| *Macrosiphum euphorbiae* | 33.789244 | 9.865123 | | Tunisia | CABI, 2019 |
| *Macrosiphum euphorbiae* | 1.373333 | 32.290275 | | Uganda | CABI, 2019 |
| *Macrosiphum euphorbiae* | -13.133898 | 27.849332 | | Zambia | CABI, 2019 |
| *Macrosiphum euphorbiae* | -19.015437 | 29.154857 | | Zimbabwe | CABI, 2019 |
| *Macrosiphum euphorbiae* | 23.684994 | 90.356331 | | Bangladesh | CABI, 2019 |
| *Macrosiphum euphorbiae* | 24.475285 | 101.343106 | Yunnan | China | CABI, 2019 |
| *Macrosiphum euphorbiae* | 33.778175 | 76.576171 | Northern Kashmir | | CABI, 2019 |
| *Macrosiphum euphorbiae* | 31.104829 | 77.17339 | Himachal Pradesh | India | Gavkare et al., 2015 |
| *Macrosiphum euphorbiae* | 29.919873 | 77.306595 | Malha Mazra (Nakur) | | Kumar et al., 2019 |
| *Macrosiphum euphorbiae* | 29.968003 | 77.555207 | Saharanpur (U.P.) | | |
| *Macrosiphum euphorbiae* | -7.090911 | 107.668887 | West Java | Indonesia | Maharani et al., 2018 |

| | | | | | |
|---|---|---|---|---|---|
| *Macrosiphum euphorbiae* | 32.427908 | 53.68804 | | Iran | CABI, 2019 |
| *Macrosiphum euphorbiae* | 31.046054 | 34.85161 | | Israel | CABI, 2019 |
| *Macrosiphum euphorbiae* | 43.220325 | 142.863471 | Hokkaido | Japan | CABI, 2019 |
| *Macrosiphum euphorbiae* | 30.585164 | 36.238414 | | Jordan | CABI, 2019 |
| *Macrosiphum euphorbiae* | 33.854721 | 35.862284 | | Lebanon | CABI, 2019 |
| *Macrosiphum euphorbiae* | 4.210484 | 101.975766 | | Malaysia | CABI, 2019 |
| *Macrosiphum euphorbiae* | 40.417567 | 127.857846 | | North Korea | CABI, 2019 |
| *Macrosiphum euphorbiae* | 35.32471 | 75.55096 | Skardu Baltistan | | Rizvi et al., 2015 |
| *Macrosiphum euphorbiae* | 33.858373 | 73.765437 | Rawalakot | | |
| *Macrosiphum euphorbiae* | 33.846499 | 73.828673 | Khaigala | | |
| *Macrosiphum euphorbiae* | 33.869787 | 73.878293 | Alisojal | | |
| *Macrosiphum euphorbiae* | 33.892772 | 73.907953 | TauliPir/Toli Pir | | |
| *Macrosiphum euphorbiae* | 33.810028 | 73.816359 | Banjosa | Pakistan | Amin et al., 2017 |
| *Macrosiphum euphorbiae* | 33.766997 | 73.89483 | Hajira | | |
| *Macrosiphum euphorbiae* | 33.81719 | 73.975657 | Abbaspur | | |
| *Macrosiphum euphorbiae* | 33.979395 | 73.777203 | Bagh | | |
| *Macrosiphum euphorbiae* | 33.948842 | 74.07701 | Haveli | | |
| *Macrosiphum euphorbiae* | 33.706041 | 73.724249 | Sudhnoti | | |
| *Macrosiphum euphorbiae* | 24.064053 | 45.736215 | | Saudi Arabia | CABI, 2019 |
| *Macrosiphum euphorbiae* | 35.907755 | 127.766919 | | South Korea | CABI, 2019 |
| *Macrosiphum euphorbiae* | 7.873054 | 80.771797 | | Sri Lanka | CABI, 2019 |
| *Macrosiphum euphorbiae* | 34.802078 | 38.99682 | | Syria | CABI, 2019 |
| *Macrosiphum euphorbiae* | 40.654895 | 29.284186 | Yalova | | |
| *Macrosiphum euphorbiae* | 40.662748 | 29.315346 | Çiftlikköy | Turkey | CABI, 2019 |
| *Macrosiphum euphorbiae* | 40.51757 | 28.882981 | Armutlu | | |
| *Macrosiphum euphorbiae* | 47.516231 | 14.550072 | | Austria | CABI, 2019 |
| *Macrosiphum euphorbiae* | 50.503887 | 4.469936 | | Belgium | CABI, 2019 |
| *Macrosiphum euphorbiae* | 42.733884 | 25.48583 | | Bulgaria | CABI, 2019 |
| *Macrosiphum euphorbiae* | 45.1 | 15.2 | | Croatia | CABI, 2019 |
| *Macrosiphum euphorbiae* | 35.126413 | 33.429859 | | Cyprus | CABI, 2019 |
| *Macrosiphum euphorbiae* | 49.817492 | 15.472962 | | Czechoslovakia | CABI, 2019 |

| | | | | | |
|---|---|---|---|---|---|
| *Macrosiphum euphorbiae* | 56.26392 | 9.501785 | | Denmark | CABI, 2019 |
| *Macrosiphum euphorbiae* | 61.924111 | 25.74815 | | Finland | CABI, 2019 |
| *Macrosiphum euphorbiae* | 46.227638 | 2.213749 | | France | CABI, 2019 |
| *Macrosiphum euphorbiae* | 51.165692 | 10.451526 | | Germany | CABI, 2019 |
| *Macrosiphum euphorbiae* | 38.322579 | 23.320431 | Thiva | Greece | Lykouressis, 2017 |
| *Macrosiphum euphorbiae* | 47.162494 | 19.503304 | | Hungary | CABI, 2019 |
| *Macrosiphum euphorbiae* | 64.963051 | -19.020835 | | Iceland | CABI, 2019 |
| *Macrosiphum euphorbiae* | 53.277639 | -7.967972 | | Ireland | CABI, 2019 |
| *Macrosiphum euphorbiae* | 40.120875 | 9.012893 | Sardinia | Italy | CABI, 2019 |
| *Macrosiphum euphorbiae* | 49.713447 | 6.130759 | | Luxembourg | CABI, 2019 |
| *Macrosiphum euphorbiae* | 35.937496 | 14.375416 | | Malta | CABI, 2019 |
| *Macrosiphum euphorbiae* | 42.708678 | 19.37439 | | Montenegro | CABI, 2019 |
| *Macrosiphum euphorbiae* | 52.132633 | 5.291266 | | Netherlands | CABI, 2019 |
| *Macrosiphum euphorbiae* | 60.472024 | 8.468944 | | Norway | CABI, 2019 |
| *Macrosiphum euphorbiae* | 51.919439 | 19.145136 | | Poland | CABI, 2019 |
| *Macrosiphum euphorbiae* | 37.764952 | -25.595707 | Azores | Portugal | CABI, 2019 |
| *Macrosiphum euphorbiae* | 32.7415 | -16.9762 | Madeira | | |
| *Macrosiphum euphorbiae* | 45.943161 | 24.966762 | | Romania | CABI, 2019 |
| *Macrosiphum euphorbiae* | 61.524005 | 105.318756 | | Russia | CABI, 2019 |
| *Macrosiphum euphorbiae* | 44.01652 | 21.005859 | | Serbia | CABI, 2019 |
| *Macrosiphum euphorbiae* | 42.708679 | 19.374389 | | Montenegro | CABI, 2019 |
| *Macrosiphum euphorbiae* | 48.669027 | 19.699025 | | Slovakia | CABI, 2019 |
| *Macrosiphum euphorbiae* | 28.291565 | -16.629128 | Canary Islands | Spain | CABI, 2019 |
| *Macrosiphum euphorbiae* | 60.128161 | 18.643499 | | Sweden | CABI, 2019 |
| *Macrosiphum euphorbiae* | 46.818188 | 8.227512 | | Switzerland | CABI, 2019 |
| *Macrosiphum euphorbiae* | 49.4335 | -2.3634 | Channel Islands | United Kingdom | CABI, 2019 |
| *Macrosiphum euphorbiae* | 50.626973 | -3.450557 | Starcross | | AHDB Aphid News, 2018 |
| *Macrosiphum euphorbiae* | 51.730815 | 0.430753 | Writtle | | |
| *Macrosiphum euphorbiae* | 32.3078 | -64.7505 | | Bermuda | CABI, 2019 |
| *Macrosiphum euphorbiae* | 53.76086 | -98.813875 | Manitoba | Canada | CABI, 2019 |
| *Macrosiphum euphorbiae* | 46.565318 | -66.461919 | New Brunswick | | |

| | | | | | |
|---|---|---|---|---|---|
| *Macrosiphum euphorbiae* | 52.939916 | -73.549138 | Quebec | | |
| *Macrosiphum euphorbiae* | 21.521757 | -77.781166 | | Cuba | CABI, 2019 |
| *Macrosiphum euphorbiae* | 18.109581 | -77.297508 | | Jamaica | CABI, 2019 |
| *Macrosiphum euphorbiae* | 21.533554 | -100.842997 | | Mexico | CABI, 2019 |
| *Macrosiphum euphorbiae* | 18.220833 | -66.590149 | | Puerto Rico | CABI, 2019 |
| *Macrosiphum euphorbiae* | 64.200844 | -149.493669 | Alaska | | |
| *Macrosiphum euphorbiae* | 36.778263 | -119.417934 | California | | |
| *Macrosiphum euphorbiae* | 39.550051 | -105.782069 | Colorado | | |
| *Macrosiphum euphorbiae* | 27.664829 | -81.515754 | Florida | | |
| *Macrosiphum euphorbiae* | 32.165622 | -82.900075 | Georgia | | |
| *Macrosiphum euphorbiae* | 19.896774 | -155.582789 | Hawaii | | |
| *Macrosiphum euphorbiae* | 44.068205 | -114.742042 | Idaho | | |
| *Macrosiphum euphorbiae* | 44.85 | -69.18 | Maine | | |
| *Macrosiphum euphorbiae* | 39.045755 | -76.641271 | Maryland | | |
| *Macrosiphum euphorbiae* | 42.40721 | -71.382437 | Massachusetts | USA | CABI, 2019 |
| *Macrosiphum euphorbiae* | 46.729552 | -94.685899 | Minnesota | | |
| *Macrosiphum euphorbiae* | 46.879681 | -110.362565 | Montana | | |
| *Macrosiphum euphorbiae* | 40.712776 | -74.005973 | New York | | |
| *Macrosiphum euphorbiae* | 35.759572 | -79.019299 | North Carolina | | |
| *Macrosiphum euphorbiae* | 40.417287 | -82.907123 | Ohio | | |
| *Macrosiphum euphorbiae* | 43.804133 | -120.554202 | Oregon | | |
| *Macrosiphum euphorbiae* | 33.836081 | 81.163725 | South Carolina | | |
| *Macrosiphum euphorbiae* | 39.32098 | -111.093731 | Utah | | |
| *Macrosiphum euphorbiae* | 47.751073 | -120.740137 | Washington | | |
| *Macrosiphum euphorbiae* | 43.075968 | -107.290283 | Wyoming | | |
| *Macrosiphum euphorbiae* | -35.000942 | 117.83925 | Albany | | |
| *Macrosiphum euphorbiae* | -35.25 | 149.1166992 | Australian Capital Territory | | |
| *Macrosiphum euphorbiae* | -34.96810913 | 138.6311951 | Urrbrae, Waite Campus | Australia | Atlas of living Australia |
| *Macrosiphum euphorbiae* | -34.93333435 | 138.6000061 | Adelaide | | |
| *Macrosiphum euphorbiae* | -46.9 | 37.75 | | | |
| *Macrosiphum euphorbiae* | -21.262263 | 165.304049 | | New Caledonia | CABI, 2019 |

| | | | | | |
|---|---|---|---|---|---|
| *Macrosiphum euphorbiae* | -41.973 | 173.071 | | New Zealand | CABI, 2019 |
| *Macrosiphum euphorbiae* | -38.416109 | -63.616665 | | Argentina | CABI, 2019 |
| *Macrosiphum euphorbiae* | -16.290153 | -63.588653 | | Bolivia | CABI, 2019 |
| *Macrosiphum euphorbiae* | -18.512178 | -44.55503 | Minas Gerais | Brazil | CABI, 2019 |
| *Macrosiphum euphorbiae* | -25.252088 | -52.021541 | Parana | | |
| *Macrosiphum euphorbiae* | -35.675151 | -71.542977 | | Chile | CABI, 2019 |
| *Macrosiphum euphorbiae* | 4.570868 | -74.297333 | | Colombia | CABI, 2019 |
| *Macrosiphum euphorbiae* | -1.831239 | -78.183406 | | Ecuador | CABI, 2019 |
| *Macrosiphum euphorbiae* | -51.796252 | -59.523612 | | Falkland Islands | CABI, 2019 |
| *Macrosiphum euphorbiae* | -9.189966 | -75.015152 | | Peru | CABI, 2019 |
| *Macrosiphum euphorbiae* | -32.52278 | -55.765836 | | Uruguay | CABI, 2019 |
| *Macrosiphum euphorbiae* | 6.423751 | -66.589729 | | Venezuela | CABI, 2019 |
| *Macrosiphum euphorbiae* | 33.886917 | 9.537499 | | Tunisian | Raboudi et al., 2012 |
| *Macrosiphum euphorbiae* | -25.747868 | 28.229271 | Pretoria | South Africa | Muller, 2016 |

Supplementary Table S6. Occurrence data of *Viteus vitifoliae*

| | Latitude | Longitude | Location | Nation | Reference |
|---|---|---|---|---|---|
| *Viteus vitifoliae* | 28.033885 | 1.659626 | | Algeria | EPPO (2014) |
| *Viteus vitifoliae* | 31.791701 | -7.09262 | | Morocco | EPPO (2014) |
| *Viteus vitifoliae* | -30.559488 | 22.937501 | | South Africa | EPPO (2014) |
| *Viteus vitifoliae* | 33.886918 | 9.537498 | | Tunisia | EPPO (2014) |
| *Viteus vitifoliae* | -19.015437 | 29.154857 | | Zimbabwe | EPPO (2014) |
| *Viteus vitifoliae* | | | | Asia | |
| *Viteus vitifoliae* | 40.0691 | 45.038188 | | Armenia | EPPO (2014) |
| *Viteus vitifoliae* | 40.143104 | 47.576927 | | Azerbaijan | EPPO (2014) |
| *Viteus vitifoliae* | 27.625299 | 111.856858 | Hunan | China | EPPO (2014); IPPC (2008) |
| *Viteus vitifoliae* | 41.943655 | 122.529036 | Liaoning | | EPPO (2014) |
| *Viteus vitifoliae* | 35.893953 | 117.924899 | Shandong | | EPPO (2014) |
| *Viteus vitifoliae* | 31.230416 | 121.473701 | Shanghai | | EPPO (2014) |
| *Viteus vitifoliae* | 42.315405 | 43.356894 | | Georgia | EPPO (2014) |
| *Viteus vitifoliae* | 33.778175 | 76.576171 | Jammu and Kashmir | India | EPPO (2014) |
| *Viteus vitifoliae* | -0.789275 | 113.921326 | | Indonesia | EPPO (2014) |
| *Viteus vitifoliae* | 31.046051 | 34.851614 | | Israel | EPPO (2014) |
| *Viteus vitifoliae* | 43.220327 | 142.863474 | Hokkaido | Japan | EPPO (2014) |
| *Viteus vitifoliae* | 36.170001 | 138.039998 | Honshu | | EPPO (2014) |
| *Viteus vitifoliae* | 32.589999 | 130.8 | Kyushu | | EPPO (2014) |
| *Viteus vitifoliae* | 33.743224 | 133.637532 | Shikoku | | EPPO (2014) |
| *Viteus vitifoliae* | 30.585164 | 36.238414 | | Jordan | EPPO (2014) |
| *Viteus vitifoliae* | 48.019571 | 66.923687 | | Kazakhstan | EPPO (2014) |
| *Viteus vitifoliae* | 33.854721 | 35.862285 | | Lebanon | EPPO (2014) |
| *Viteus vitifoliae* | 40.339853 | 127.510094 | | North Korea | EPPO (2014) |
| *Viteus vitifoliae* | 35.907755 | 127.766919 | | South Korea | EPPO (2014) |
| *Viteus vitifoliae* | 34.802075 | 38.996818 | | Syria | EPPO (2014) |
| *Viteus vitifoliae* | 38.963745 | 35.243323 | | Turkey | EPPO (2014); Gözel and Gözel (2014) |
| *Viteus vitifoliae* | 41.377494 | 64.585262 | | Uzbekistan | EPPO (2014) |
| *Viteus vitifoliae* | | | | Europe | |

| | | | | | |
|---|---|---|---|---|---|
| *Viteus vitifoliae* | 47.516231 | 14.550072 | | Austria | EPPO (2014) |
| *Viteus vitifoliae* | 50.503887 | 4.469936 | | Belgium | EPPO (2014) |
| *Viteus vitifoliae* | 43.915885 | 17.679076 | | Bosnia and Herzegovina | EPPO (2014) |
| *Viteus vitifoliae* | 42.733882 | 25.485831 | | Bulgaria | EPPO (2014) |
| *Viteus vitifoliae* | 45.1 | 15.2 | | Croatia | EPPO (2014) |
| *Viteus vitifoliae* | 35.126413 | 33.429859 | | Cyprus | EPPO (2014) |
| *Viteus vitifoliae* | 49.817491 | 15.472962 | | Czechia | EPPO (2014) |
| *Viteus vitifoliae* | 58.595271 | 25.013608 | | Estonia | EPPO (2014) |
| *Viteus vitifoliae* | 61.924108 | 25.748151 | | Finland | EPPO (2014) |
| *Viteus vitifoliae* | 42.039604 | 9.012892 | Corsica | France | EPPO (2014) |
| *Viteus vitifoliae* | 51.165692 | 10.451527 | | Germany | EPPO (2014) |
| *Viteus vitifoliae* | 39.074201 | 21.824312 | | Greece | EPPO (2014) |
| *Viteus vitifoliae* | 49.456945 | -2.587384 | | Guernsey | EPPO (2014) |
| *Viteus vitifoliae* | 47.162495 | 19.503304 | | Hungary | EPPO (2014) |
| *Viteus vitifoliae* | 40.120875 | 9.012893 | Sardinia | Italy | EPPO (2014) |
| *Viteus vitifoliae* | 37.599994 | 14.015356 | Sicily | | EPPO (2014) |
| *Viteus vitifoliae* | 56.879635 | 24.603189 | | Latvia | EPPO (2014) |
| *Viteus vitifoliae* | 49.815273 | 6.129583 | | Luxembourg | EPPO (2014) |
| *Viteus vitifoliae* | 35.937496 | 14.375416 | | Malta | EPPO (2014) |
| *Viteus vitifoliae* | 47.411633 | 28.369886 | | Moldova | EPPO (2014) |
| *Viteus vitifoliae* | 42.708678 | 19.37439 | | Montenegro | EPPO (2014) |
| *Viteus vitifoliae* | 52.132633 | 5.291266 | | Netherlands | EPPO (2014) |
| *Viteus vitifoliae* | 41.608635 | 21.745275 | | North Macedonia | EPPO (2014) |
| *Viteus vitifoliae* | 60.472023 | 8.468946 | | Norway | EPPO (2014) |
| *Viteus vitifoliae* | 51.919439 | 19.145136 | | Poland | EPPO (2011); EPPO (2014) |
| *Viteus vitifoliae* | 37.7841 | -25.534907 | Azores | Portugal | EPPO (2014) |
| *Viteus vitifoliae* | 32.760707 | -16.959471 | Madeira | | EPPO (2014) |
| *Viteus vitifoliae* | 45.943161 | 24.966762 | | Romania | EPPO (2014) |
| *Viteus vitifoliae* | 47.335244 | 43.425747 | Southern Russia | Russia | EPPO (2014) |
| *Viteus vitifoliae* | 44.016521 | 21.005857 | | Serbia | EPPO (2014) |
| *Viteus vitifoliae* | 48.669026 | 19.699025 | | Slovakia | EPPO (2014) |

| | | | | | |
|---|---|---|---|---|---|
| *Viteus vitifoliae* | 46.151241 | 14.995463 | | Slovenia | EPPO (2014) |
| *Viteus vitifoliae* | 39.52718 | 2.848734 | Balearic Islands | Spain | EPPO (2014) |
| *Viteus vitifoliae* | 46.818188 | 8.227512 | | Switzerland | EPPO (2014) |
| *Viteus vitifoliae* | 48.379432 | 31.165582 | | Ukraine | EPPO (2014) |
| *Viteus vitifoliae* | 55.378051 | -3.435974 | | United Kingdom | EPPO (2014); |
| *Viteus vitifoliae* | | | | North America | |
| *Viteus vitifoliae* | 32.3078 | -64.7505 | | Bermuda | EPPO (2014) |
| *Viteus vitifoliae* | 53.726671 | -127.647621 | British Columbia | | EPPO (2014) |
| *Viteus vitifoliae* | 53.760862 | -98.813877 | Manitoba | Canada | EPPO (2014) |
| *Viteus vitifoliae* | 51.253776 | 85.323213 | Ontario | | EPPO (2014) |
| *Viteus vitifoliae* | 21.533555 | -100.842996 | | Mexico | EPPO (2014) |
| *Viteus vitifoliae* | 8.76018 | -80.150998 | | Panama | EPPO (2014) |
| *Viteus vitifoliae* | 34.048928 | -111.093732 | Arizona | | EPPO (2014) |
| *Viteus vitifoliae* | 35.20105 | -91.831834 | Arkansas | | EPPO (2014) |
| *Viteus vitifoliae* | 36.778264 | -119.417932 | California | | EPPO (2014) |
| *Viteus vitifoliae* | 41.603221 | -73.087749 | Connecticut | | EPPO (2014) |
| *Viteus vitifoliae* | 34.51994 | -105.870089 | New Mexico | | EPPO (2014) |
| *Viteus vitifoliae* | 40.712776 | -74.005973 | New York | United States | EPPO (2014) |
| *Viteus vitifoliae* | 40.417286 | -82.907123 | Ohio | | EPPO (2014) |
| *Viteus vitifoliae* | 43.804133 | -120.554201 | Oregon | | EPPO (2014) |
| *Viteus vitifoliae* | 41.203322 | -77.194525 | Pennsylvania | | Downie et al. (2001) |
| *Viteus vitifoliae* | 31.9686 | -99.901814 | Texas | | EPPO (2014) |
| *Viteus vitifoliae* | 47.751073 | -120.740137 | Washington | | EPPO (2014) |
| *Viteus vitifoliae* | | | | Oceania | |
| *Viteus vitifoliae* | -31.253218 | 146.9211 | New South Wales | | CABI (Undated) |
| *Viteus vitifoliae* | -20.91757 | 142.702805 | Queensland | Australia | EPPO (2014) |
| *Viteus vitifoliae* | -30.000234 | 136.209158 | South Australia | | EPPO (2014) |
| *Viteus vitifoliae* | -37.471308 | 144.785152 | Victoria | | EPPO (2014) |
| *Viteus vitifoliae* | -40.900554 | 174.885983 | | New Zealand | EPPO (2014) |
| *Viteus vitifoliae* | | | | South America | |
| *Viteus vitifoliae* | -38.416092 | -63.616683 | | Argentina | EPPO (2014) |

| | | | | | |
|---|---|---|---|---|---|
| *Viteus vitifoliae* | -16.290153 | -63.588653 | | Bolivia | EPPO (2014) |
| *Viteus vitifoliae* | -12.579739 | -41.700725 | Bahia | Brazil | EPPO (2014) |
| *Viteus vitifoliae* | -18.512179 | -44.555034 | Minas Gerais | | EPPO (2014) |
| *Viteus vitifoliae* | -25.252088 | -52.021541 | Parana | | EPPO (2014) |
| *Viteus vitifoliae* | -8.813718 | -36.954107 | Pernambuco | | EPPO (2014) |
| *Viteus vitifoliae* | -22.906848 | -43.172897 | Rio de Janeiro | | EPPO (2014) |
| *Viteus vitifoliae* | -30.034617 | -51.2177 | Rio Grande do Sul | | EPPO (2014) |
| *Viteus vitifoliae* | -27.24234 | -50.218855 | Santa Catarina | | EPPO (2014) |
| *Viteus vitifoliae* | -23.55052 | -46.633309 | Sao Paulo | | EPPO (2014) |
| *Viteus vitifoliae* | 4.570868 | -74.297333 | | Colombia | EPPO (2014) |
| *Viteus vitifoliae* | -9.189969 | -75.015152 | | Peru | EPPO (2014) |
| *Viteus vitifoliae* | -32.52278 | -55.765836 | | Uruguay | EPPO (2014) |
| *Viteus vitifoliae* | 6.423751 | -66.589729 | | Venezuela | EPPO (2014) |
